# Parental Expression Variation of Small RNAs Is Negatively Correlated with Grain Yield Heterosis in a Maize Breeding Population

**DOI:** 10.3389/fpls.2018.00013

**Published:** 2018-01-30

**Authors:** Felix Seifert, Alexander Thiemann, Robert Grant-Downton, Susanne Edelmann, Dominika Rybka, Tobias A. Schrag, Matthias Frisch, Hugh G. Dickinson, Albrecht E. Melchinger, Stefan Scholten

**Affiliations:** ^1^Biocenter Klein Flottbek, University of Hamburg, Hamburg, Germany; ^2^Department of Plant Sciences, University of Oxford, Oxford, United Kingdom; ^3^Institute for Plant Breeding, Seed Science and Population Genetics, University of Hohenheim, Stuttgart, Germany; ^4^Institute of Agronomy and Plant Breeding II, Justus-Liebig University, Giessen, Germany

**Keywords:** heterosis, non-coding RNAs, hybrid breeding, heterotic groups, genome evolution

## Abstract

Heterosis refers to a quantitative phenomenon in which F1 hybrid trait values exceed the mean of the parental values in a positive direction. Generally, it is dependent on a high degree of heterozygosity, which is maintained in hybrid breeding by developing parental lines in separate, genetically distinct heterotic groups. The mobility of small RNAs (sRNAs) that mediate epigenetic regulation of gene expression renders them promising candidates for modulating the action of combined diverse genomes in *trans*–and evidence already indicates their contribution to transgressive phenotypes. By sequencing small RNA libraries of a panel of 21 maize parental inbred lines we found a low overlap of 35% between the sRNA populations from both distinct heterotic groups. Surprisingly, in contrast to genetic or gene expression variation, parental sRNA expression variation is negatively correlated with grain yield (GY) heterosis. Among 0.595 million expressed sRNAs, we identified 9,767, predominantly 22- and 24-nt long sRNAs, which showed an association of their differential expression between parental lines and GY heterosis of the respective hybrids. Of these sRNAs, 3,485 or 6,282 showed an association with high or low GY heterosis, respectively, thus the low heterosis associated group prevailing at 64%. The heterosis associated sRNAs map more frequently to genes that show differential expression between parental lines than reference sets. Together these findings suggest that trans-chromosomal actions of sRNAs in hybrids might add up to a negative contribution in heterosis formation, mediated by unfavorable gene expression regulation. We further revealed an exclusive accumulation of 22-nt sRNAs that are associated with low GY heterosis in pericentromeric genomic regions. That recombinational suppression led to this enrichment is indicated by its close correlation with low recombination rates. The existence of this enrichment, which we hypothesize resulted from the separated breeding of inbred lines within heterotic groups, may have implications for hybrid breeding strategies addressing the recombinational constraints characteristic of complex crop genomes.

## Introduction

Heterosis describes a quantitative phenomenon in which hybrid trait values exceed the parental values in a positive direction (Shull, 1908). This increased vigor of heterozygous F1 plants is extensively exploited by modern hybrid breeding and has provided substantial gains for agronomically important traits. Heterosis has been exploited to an outstanding degree in maize (Duvick, 2001; Schnable and Springer, 2013) and is increasingly utilized in other important crops to meet growing world demands for food and bioenergy (Longin et al., 2012).

Despite its importance, the molecular basis of heterosis has remained elusive, although it is widely accepted that it results from the combined action of diverged genomes, and that a range of genetic mechanisms are involved (for review, see Birchler et al., 2010; Chen, 2013; Schnable and Springer, 2013). To maintain the high degree of genetic diversity that heterosis is dependent on, in hybrid breeding programmes the parental lines are developed in separate, genetically distinct “heterotic groups.” In nature, the combination of genetically diverged genomes has both positive and negative phenotypic outcomes. The effect of outcrossing on inbred populations can lead to enhanced fitness and genetic rescue (Ingvarsson, 2001). Interspecific hybridization can also lead to hybrid vigor but phenotypic changes with detrimental effects on fitness, ranging from subtle to severe, are often evident (e.g., Dobzhansky, 1937; Stebbins, 1950; Grant, 1971). Whereas in breeding contexts better-parent heterosis—which measures the superiority of the hybrid relative to the *better* parent—is the metric of interest, for quantitative genetic analysis of the phenomenon mid-parental heterosis is the relevant value as this measures the deviation of the hybrid relative to the mean of the two parents, quantifying heterosis as the difference in the performance of the hybrid relative to the average of the inbred parents (Kaeppler, 2012).

Epigenetic variations are increasingly recognized as important components of crop yield (Rodríguez López and Wilkinson, 2015) and various studies have shown that there may be an epigenetic component to the generation of heterosis in plants (reviewed in Ng et al., 2012; Chen, 2013; Groszmann et al., 2013). The recent demonstration of heterosis in the absence of genetic diversity by crosses of epigenetic inbred lines (Dapp et al., 2015), strongly support the contribution of epigenetic components to heterosis in addition to genetic factors. Hybrids of *Arabidopsis* ecotypes and rice subspecies showed substantial variations at the level of DNA methylation, histone modifications and sRNAs (He et al., 2010; Shen et al., 2012). These hybrid-specific epigenetic states are likely to be established early after fertilization. In maize, overdominant gene expression patterns signify that interactions of the parental genomes have already been established in early embryos, a developmental stage when growth heterosis is also already very evident (Meyer and Scholten, 2007; Meyer et al., 2007). In *Arabidopsis*, epigenetic regulatory pathways exhibit especially high activity during embryogenesis, and epigenetic patterns established during early zygotic development are maintained in the adult plant (Jullien et al., 2012).

For the epigenetic modulation of diverse genomes in hybrids, sRNAs are promising candidates. They typically regulate gene expression by post-transcriptional RNA interference in the cytoplasm and in the nucleus at the transcriptional level through directing epigenetic modifications, and importantly have the ability to act in *trans* (Castel and Martienssen, 2013). Numerous small RNAs are transgressively expressed in interspecific tomato hybrids, which coincide with hypermethylation and gene expression changes (Shivaprasad et al., 2011). In reciprocal maize hybrids, reduction of 24-nt sRNAs from repetitive DNA as well as changes in 21-22-nt sRNAs was observed (Barber et al., 2012). Collectively, the trend was for down-regulation of sRNA production in hybrids of various plant species compared to their parents (He et al., 2010, 2013; Groszmann et al., 2011; Kenan-Eichler et al., 2011; Barber et al., 2012; Li et al., 2012; Shen et al., 2012). Although the precise mechanisms behind the epigenetic changes remain unknown, the consensus is that a component of heterosis is due to sRNA-influenced changes in gene expression. However, correlations of sRNA expression pattern with heterotic traits could not be established yet, because the numbers of inbred line or ecotype combinations analyzed were limited and the establishment of statistically valid associations is highly dependent on the number of genotypes that are measured for performance.

To facilitate an association study that addresses the relation between the phenomenon heterosis and parental sRNAs expression in maize, we employed a factorial mating scheme involving 98 F1 hybrid combinations of 14 and 7 parental inbred lines of two heterotic groups. All genotypes were tested for the highly heterotic trait grain yield (GY, Schrag et al., 2006) and we sequenced the sRNAs of seedlings of all inbred lines and 3 representative hybrids that exhibit low, medium, and high heterosis for GY. Our approach did not apply any functional a priori assumptions concerning sRNAs to account for the fact that the exploration of sRNA-mediated mechanisms and functions is by far complete (Borges and Martienssen, 2015), especially in large and complex cereal genomes. As a consequence, our strategy relied solely on statistically founded correlations and associations to identify sRNAs that are associated with heterosis. We deduced potential functions and genetic mechanism involvement of the sRNA sets we identified subsequently by bioinformatic characterization.

## Materials and methods

### Plant material and phenotyping

The plant material for our association study consists of 21 maize inbred lines (7 flint and 14 dent) and 98 hybrids resulting from a factorial mating scheme of the breeding program of the University of Hohenheim, Germany. The seven flint lines comprise four with European Flint background (F037, F039, F043, F047) and three with Flint/Lancaster background (L028, L035, L043). Eight of 14 dent lines have an Iowa Stiff Stalk Synthetic (S028, S036, S044, S046, S049, S050, S058, S067) and six have an Iodent background (P033, P040, P046, P048, P063, and P066). Phenotypic data were collected from field trials, for the inbred lines in 2003 and 2004 at five locations and for the hybrids in 2002 at six locations in Germany. GY field data were measured in Mg ha^−1^ adjusted to 155 g kg^−1^ grain moisture [27,32]. For sRNA sequencing all inbred lines, the inbred line B73 as a reference, and three inter-pool hybrids, chosen to cover a high (123.73%, P033xF047), an intermediate (84.60%, S028xF039) and a low (72.51%, S028xL024) MPH for GY level, were grown in five biological replicates under controlled conditions (25°C, 16 h day, 8 h night, 70% air humidity) for 7 days. Whole plants were flash-frozen in liquid nitrogen and individuals of the same genotypes were pooled before RNA isolation.

### RNA preparation and sequencing

Total RNA was isolated using the mirVana miRNA Isolation Kit (Life Technologies Corp., Carlsbad, USA). The quality of the RNA was tested by photometrical and gelelectrophoretic analyses. Sequencing library preparation with TruSeq Kits and HiSeq 2000 sequencing (Illumina Inc, San Diego, USA) were performed by Eurofins MWG GmbH (Ebersberg, Germany) or LGC Genomics GmbH (Berlin, Germany). The sRNA libraries were indexed with barcodes and up to four samples were sequenced per lane. All sequence data has been deposited in the GEO under accession number GSE51662.

### Processing and normalization of sRNA sequencing data

The raw sequencing data were processed by custom Java-programs and R-scripts. After adapter removal the reads were trimmed to 99.9% sequencing quality. All sequence reads ranging from 15- to 40-nt were retained, redundant reads were merged to obtain raw read counts.

The raw read counts of all 21 inbred lines, 3 hybrids, and the reference line B73were quantile normalized (Bolstad et al., 2003) with a modification preventing the allocation of read counts to sequence tags not expressed in the sample. The normalized read counts were finally scaled to one million reads per library resulting in read counts per million quantile normalized (rpmqn) reads allowing for a comparison of sequencing libraries with varying sequencing depths.

### sRNA differential expression analysis

The differential expression state *x*_*s*_ (*x*_*s*_ = 1 for differential expression and *x*_*s*_ = 0 otherwise) of a the sRNA s is defined for two inbred lines i and j with read counts *c*_*i*_ and *c*_*j*_ with *c*_*h*_ = max(*c*_*i*_, *c*_*j*_) and *c*_*l*_ = min(*c*_*i*_, *c*_*j*_) as well as the predefined parameters for minimal expression *c*_*min*_ and fold-change *f*_*c*_ as follows:

(1) $$x_{s}\text{ = }\{\begin{array}{ll} 1 & if\;c_{t}>c_{min}\Lambda \;c_{h}>=c_{t}\cdot f_{c}\\ 1 & if\;c_{t}=0\Lambda \;c_{h}>=c_{min}\cdot f_{c}\\ 0 & \;otherwise \end{array}$$

Thus the sRNA is differentially expressed if the ratio of the inbred parent with higher read counts *c*_*h*_ exceeds the inbred with lower read counts *c*_*l*_ by the fold-change *f*_*c*_. The minimal expression *c*_*min*_ avoids overestimation of differential expression for marginal read counts and facilitates differential expression states in the case of absence of the sRNA in one parent. The minimal expression was defined as *c*_*min*_ = 0.5 rpmqn for all analyses throughout this study. The fold-change for differential expression analyses was defined as *f*_*c*_ = 2.

### Inbred line sRNA population diversity and discriminative power

The sRNA population diversity was determined at the defined minimal expression *c*_*min*_ = 0.5 rpmqn by calculating the fraction of specific and intersecting read sets for inbred lines of both heterotic groups. For the summary of heterotic group specific sRNAs all sRNA with inconsistent presence/absence expression patterns in different inbred line combinations were excluded. The discriminative power of differentially expressed sRNAs for separation of inbred lines according to their genetic distance into their heterotic groups was determined by calculation of the binary distance for all inbred line pairings. The binary distance was calculated based on sRNA differential expression by formula 1 with minimal read count *c*_*min*_ = 0.5 and fold-change *f*_*c*_ = 2 for each of all n sRNAs for the two inbred lines *i* and *j* as follows:

(2) $$D_{b}\left(i,j\right)=\sqrt{\frac{1}{n}\sum_{s=1}^{n}x_{s}}$$

The grouping of the inbred lines was performed by the first three components of a principal component analysis (PCA) based on their binary distance.

### Comparison of sRNA data with SNP data and mRNA data

SNP-data were generated with the Illumina MaizeSNP50 chip (Frascaroli et al., 2013); mRNA data were generated by microarray hybridizations (Thiemann et al., 2010). The euclidean distances *D*_*e*_ Equation (3) were calculated for all three data types as the sum of the absolute expression differences between the lines *i* and *j*, with *d*_*s*_*(i,j)* being the expression difference for a specific sRNA or mRNA Equation (4) or SNP Equation (5).

(3) $$D_{e}\left(i,j\right)=\sqrt{\sum_{s=1}^{n}d_{s}{\left(i,j\right)}^{2}}$$

The expression difference *d*_*s*_ for sRNA and mRNA expression data *c*_*s*_ between the lines *i* and *j* is calculated as follows:

(4) $$d_{s}\left(i,j\right)=\left(c_{s}\left(i\right)=c_{s}\left(j\right)\right)$$

The difference *d*_*s*_ for SNP data with *c*_*s*_ being the actual sequence between the lines *i* and *j* is calculated as follows:

(5) $$d_{s}\left(i,j\right)\text{ = }\{\begin{array}{lll} 0 & if & c_{s}\left(i\right)\neq c_{s}\left(j\right)\\ 1 & if & c_{s}\left(i\right)=c_{s}\left(j\right) \end{array}$$

### sRNA trait association analysis

Association of sRNA expression to MPH for GY and BPH for GY (data not shown) were established analogous to Frisch et al. (2010) by separating the hybrids into the classes of low and high trait values (*L, H*) with equal size. For each small RNA the number of hybrids with differential expression (*c*_*min*_ = 0.5, *f*_*c*_ = 2) between the inbred parents was counted for both classes *L* and *H* as *o*_*L*_ and *o*_*H*_ respectively. With the null hypothesis that differential expression occurs with the same probability for both classes, the probability *P*_*s*_ Equation (6) of an sRNA being associated to MPH for GY was estimated via the binomial distribution probability function. This function depends on the number of hybrids whose inbred lines exhibit differential expression for the given sRNA in the classes *L* and *H*:

(6) $$P_{s}=\sum_{k=k_{min}}^{n}Bin_{n,p}\left(k\right)\;with\;n=\left(o_{H}+o_{L}\right),p=0.5$$

with the parameter k_*min*_ depending on positive Equation (7) or negative Equation (8) association:

(7) $$k_{\min}=o_{L}\;if\;o_{L}>o_{H}$$

(8) $$k_{\min}=o_{H}\;if\;o_{L}<=o_{H}$$

All sRNAs with *p*-values lower than the probability threshold, adjusted for multiple testing via Benjamini-Hochberg FDR correction for *p* ≤ 0.05 (Benjamini and Hochberg, 1995), were considered as associated to MPH for GY and termed heterosis-associated sRNAs (ha-sRNAs). The certainty of the association against random artifacts was tested by permutation analyses (100 runs) of the datasets by either randomly re-assigned hybrid trait values (MPH for GY) to the hybrids or re-assignment of inbred line labels. Binary distances according to Equation (2) based on associated sRNAs were used for correlation analyses with MPH for GY.

### sRNA expression pattern analysis in hybrids

The dominance to additivity (d/a) calculation was performed for all differentially expressed sRNAs (parameters *c*_*min*_ = 0.5 and *f*_*c*_ = 2) and individually for the subsets of negatively and positively ha-sRNAs as described by Li et al. (2012).

### sRNA enrichment analyses

The significances of enrichment and depletion for MPH for GY associated sRNAs of specific sequence length were computed by bootstrap analysis with 1,000 runs. The bootstrap sets of sRNAs were composed of randomly selected sRNAs with equal set size as the reference set. The enrichment and depletion analysis of ha-sRNAs for repeat super families was performed by bootstrap analysis of all sRNAs mapping to any repeat annotation. The bootstrap analysis was performed with 1,000 runs comparing the distribution of randomly selected sRNAs with identical size distribution as the associated sRNAs with the latter.

### Mapping of sRNAs and annotation analysis

HISAT (version 2.1.0, parameters: “–all –no-spliced-alignment –sp 1000 –mp 1000 –rdg 1000,1000 –rfg 1000,1000”; Kim et al., 2015) was used to map sRNAs to reference sequences. For the distribution analysis we used 1 kbp windows along the B73 reference genome sequence (AGPv4; ftp://ftp.gramene.org/pub/gramene/CURRENT_RELEASE/fasta/zea_mays/dna/, July 2017; Jiao et al., 2017). To explore genomic features to which ha-sRNAs map, a maize genome annotation database was established. It comprise the working gene set models (version AGPv4.36; downloaded from ftp://ftp.gramene.org/pub/gramene/CURRENT_RELEASE/gff3/zea_mays/, July 2017) defining “gene” regions and the repeat annotations (version AGPv4; downloaded from ftp://ftp.gramene.org/pub/gramene/CURRENT_RELEASE/gff3/zea_mays/repeat_annotation/, July 2017) specifying all “repeat” regions. All genomic regions without gene or repeat annotation were defined as “intergenic.” The sRNAs were allowed to map to multiple annotations. If this was the case they were attributed to the conjunction of these annotations. Thus each distinct sRNA is either attributed to a single annotation or the conjunction of different annotations. The coverage of annotation types in bp per Mbp was calculated for sequential windows of the genome sequence. The spatial 2-fold enrichment for MPH for GY associated sRNAs of specific sequence lengths was computed by bootstrap analysis with 10,000 runs in sequential genome sequence windows of 1 Mbp. The bootstrap sets of sRNAs were composed of randomly selected sRNAs with equal set size as the reference set. For further characterization, all sRNAs were mapped to repeat sequences downloaded from the Maize TE database (http://www.maizetedb.org/~maize/, July 2017) without mismatches allowed. To identify known miRNAs and pre-miRNA derived ha-sRNAs or ha-sRNAs with homology to tRNA sequences the ha-sRNAs were mapped to pre-miRNA sequences from miRBase Release 21 (Griffiths-Jones et al., 2006) or to Zea mays (Version 5b.60) tRNA sequences from GtRNAdb (Chan and Lowe, 2009), respectively, without mismatches with HISAT (version 2.1.0) (Kim et al., 2015). The identification of ha-sRNAs with homology to rRNA sequences was done similarly by mapping the ha-sRNAs to Zea mays (Version 5b.60) rRNA sequences from SILVA SSU and LSU databases (release 128) (Quast et al., 2012). Recombination rates for windows of 1 Mbp of B73 AGPv44 was calculated according to Liu et al. (2009) based on the map Genetic 3 (downloaded from http://www.maizegdb.org/data_center/map?id=1203639, August 2017).

### sRNA cluster analyses

siRNA clusters were identified by merging sRNAs mapping within 200 bp to each other to the B73 reference genome (AGPv4, downloaded from ftp://ftp.gramene.org/pub/gramene/CURRENT_RELEASE/fasta/zea_mays/dna/, July 2017). The sRNA cluster expression was determined by distributing sRNA expression (rpmqn) equally to all loci by dividing the expression by number of mapping positions (repeat-normalization). All clusters with at least 5 individual sRNAs mapping to it were retained for further analyses. The association of sRNA clusters to MPH for GY was performed analogous to the sRNA trait association. The minimal expression for a cluster was set to *c*_*min*_ = 5 repnorm rpmqn and the fold-change to *f*_*c*_ = 2.

### Processing of the B73/MO17 RNA sequencing data

The RNAseq and sRNAseq data from 5 day old shoots at the coleoptilar stage of the maize inbred lines B73 and Mo17 generated by (Regulski et al., 2013) were obtained from NCBI GEO series GSE39232. The sequences were preprocessed by custom Java-programs. Concerning mRNA data, trimmed RNAseq reads were aligned to the B73 reference genome (RefGen_v4, downloaded from ftp://ftp.gramene.org/pub/gramene/CURRENT_RELEASE/fasta/zea_mays/dna/, July 2017) using Tophat v2.0.13 (Trapnell et al., 2009) with default parameters. The reads were assembled using Cufflinks v2.2.1 with the working gene set (version AGPv4.36, downloaded from http://ftp.gramene.org/pub/gramene/CURRENT_RELEASE/gff3/zea_mays/, July 2017), the results of all replicates merged using Cuffmerge v1.0.0, and differentially expressed genes were identified using Cuffdiff v2.2.1 (Trapnell et al., 2012).

### Analysis for ha-sRNA enrichment at regions of differentially expressed genes

The sRNA data of all replicates in the Regulski et al. dataset of B73 and Mo17 inbred lines (Regulski et al., 2013) were normalized to rpmqn as described above. All ha-sRNAs present in this dataset were identified and tested for differential expression based on the average of the replicates using formula (1) with parameters *c*_*min*_ = 0.5 and *f*_*c*_ = 2. The enrichment of differentially expressed ha-sRNAs was separately tested for 22- and 24-nt species from 1 kbp upstream to 1 kbp downstream the gene body of differentially expressed genes. The enrichment at regions of differentially expressed genes was tested via bootstrap analyses in 1000 iterations with equally sized sets of random sRNAs with equal size-distribution. The sRNA sequences were mapped to the B73 reference genome sequence (2RefGen_vAGPv4; http://ftp.gramene.org/pub/gramene/CURRENT_RELEASE/fasta/zea_mays/dna/, AprilJuly 2017) (Jiao et al., 2017) using Bowtie (v1.1.2, Langmead et al., 2009, parameters: “-a –v 0”). Differentially expressed genes were called from B73/Mo17 RNAseq data as described in the previous paragraph and from microarray data of the plant material used in the present study (Thiemann et al., 2010) with differential expression between at least one of the inbred line combinations.

### Data access

All sequence data described in this study has been deposited in the GEO under accession number GSE51662.

All in-house scripts described in the material and methods section were made available on GitHub: https://github.com/fseifert-uhh/srna-heterosis/.

## Results

### Heterotic groups exhibit highly diverse populations of sRNAs

**Figure 1:**
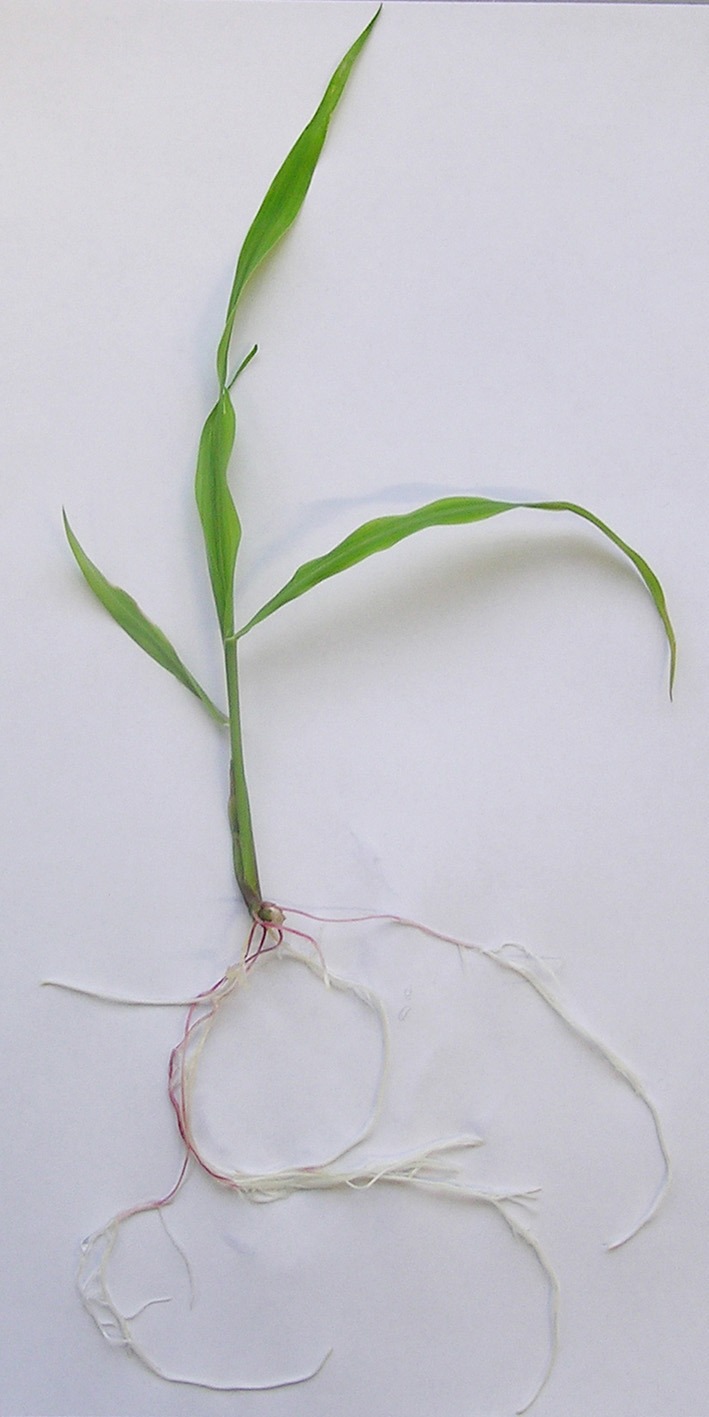
Seven-day-old maize seedling representative for the sRNA sequencing samples.

**Figure 2:**
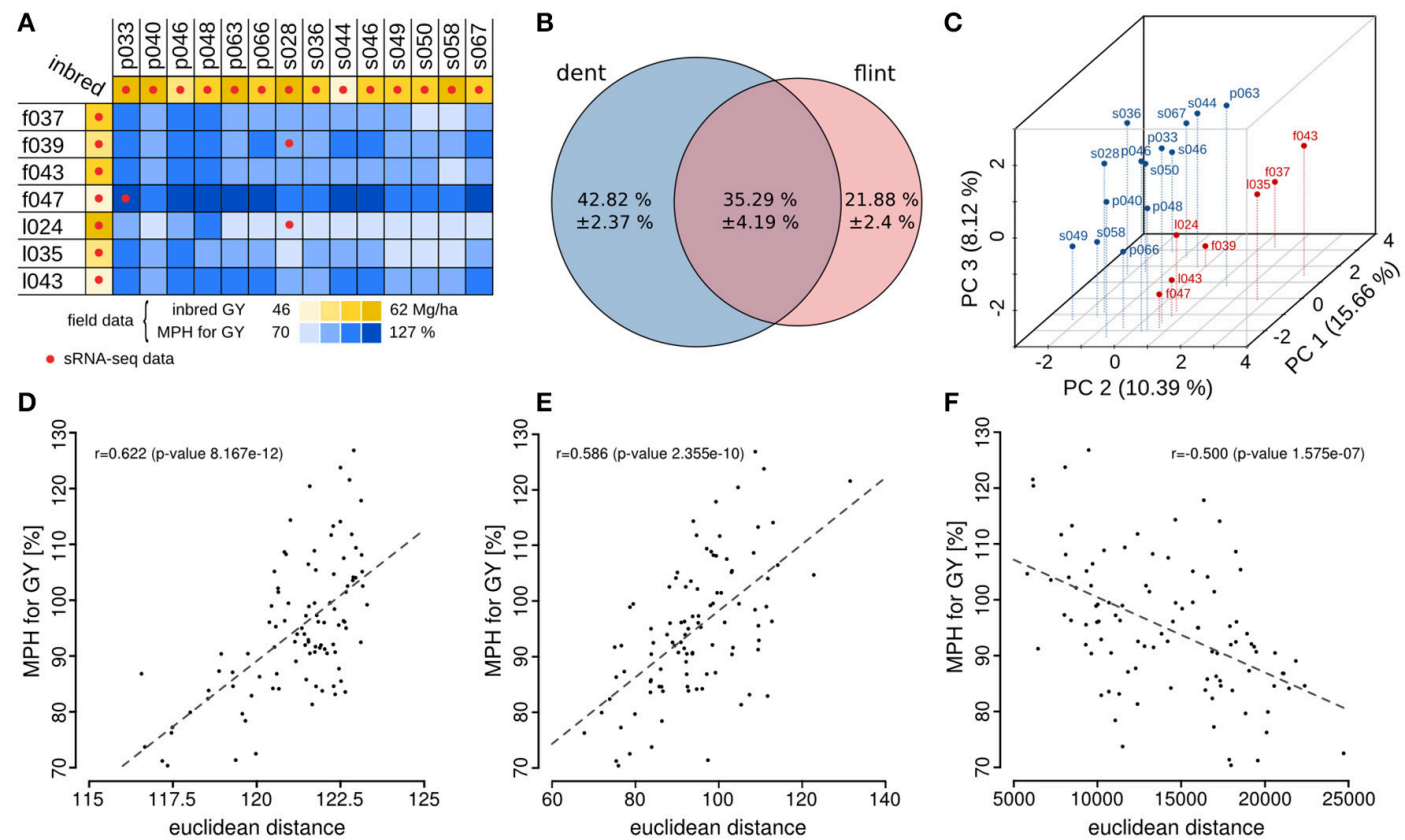
Characterization of sRNAs in a set of maize genotypes and their association with heterosis. **(A)** The 7 (Flint) and 14 (Dent) inbred line factorial crossing scheme with values for grain yield (GY) and mid-parental heterosis (MPH) for GY; the genotypes with sRNA-seq data are indicated. **(B)** Summarized sRNA-population specificity calculated from 98 inbred parent combinations of two heterotic groups showing that only a minor fraction of sRNAs is expressed in both inbred line groups. See also Supplementary Tables 3 and 4. **(C)** Separation of inbred lines via PCA based on differentially expressed sRNAs results in clustering of the heterotic groups (Dent lines in red, Flint lines in blue). **(D–F)** Correlation of genetic differences (Euclidean distance) between inbred parents with MPH for GY in the resulting hybrids based on single nucleotide polymorphism data **(D)**, mRNA expression data **(E)**, and sRNA expression data **(F)**.

We systematically explored the relationship between sRNAs in 7-day-old seedlings (Figure 1) and GY heterosis in maize, by making use of a factorial mating scheme in which parental inbred lines from two heterotic groups (Dent [14 lines] and Flint [7 lines]) were crossed to generate 98 F1 hybrids. All genotypes were tested for GY in multiple field trials and adjusted heterosis values were calculated for the hybrids (Schrag et al., 2006; Figure 2A and Supplementary File S1). Deep sequencing of sRNAs to a minimum of 12.8 million raw reads from the 7-day-old seedlings from all 21 inbred lines and three heterotic hybrids revealed between 1.3 and 4.3 million unique sRNA species of 18- to 28-nt length per genotype. In total we identified 31.1 million unique sRNAs in the 21 inbred lines and a number of 6.1 million additional unique sRNAs in three selected hybrids that are indicated in Figure 2A. Further sequencing details are shown in Supplementary File S2.

At first, we tested for the variation of sRNA expression within our inbred line population. To avoid random noise related to varying sequencing depths, we applied a minimum expression threshold of 0.5 read counts per million quantile normalized reads (rpmqn) for all further analyses. This equals to a minimum of 4 reads in any library given that we obtained at least 9.1 million reads after filtering. Of the 0.595 million sRNAs expressed under these conditions within all 21 inbred lines, we found only 35.3% with common expression in both heterotic groups. Conversely, most sRNAs from pairs of inbred lines from the two heterotic groups proved to be specific to one or other inbred line, revealing a surprisingly high level of inter-group variability in sRNA populations (Figure 2B and Supplementary File S3). To test whether this effect is dependent on the expression threshold applied, we analyzed the overlaps between inbred parents and heterotic groups with higher expression thresholds again. Although the overlap increased with higher expression thresholds, a considerable fraction of sRNA stayed group specific. At a ten-fold increased expression threshold, for example, still 16.1 and 28.4% of the sRNA population showed Flint or Dent specific expression, respectively (Supplementary File S4). These results confirm a high level of diversity in sRNA populations between the heterotic groups.

A principal component analysis (PCA), based on two-fold differentially expressed sRNAs between at least one pair of inbred lines, reliably separated all inbred lines into their respective heterotic group considering the variance covered by the first three principal components (Figure 2C). Both the high variability of sRNA expression between the heterotic groups and the effective sRNA-based grouping of the germplasm technically indicate a strong link between heterosis and expression variation of sRNAs.

### Parental variation of expressed sRNAs correlates negatively with heterosis

Genetic variation between inbred lines is usually weak but positively correlated with the heterosis of their hybrids (Melchinger, 1999). Also, variation in messenger RNA expression between parental lines is positively correlated with heterosis (Guo et al., 2006; Frisch et al., 2010). To facilitate the comparisons of different data types under identical conditions, we used distance measures, which essentially accumulate all the existing differences between inbred line pairs to a distinct value. We tested for correlations with the heterotic response of hybrids in terms of mid-parent heterosis (MPH) for grain yield (GY) with these global distance measures between the corresponding parental lines based on 50 k Illumina array single nucleotide polymorphism (SNP) data (Frascaroli et al., 2013), messenger RNA expression data (Frisch et al., 2010; Thiemann et al., 2010), and our sRNA expression data. All data were collected from the same genotypes of our factorial mating scheme and we found significant correlations of comparable strength with MPH for GY for all three data types. Unexpectedly, while correlations between MPH for GY and SNP-based genetic differences (Figure 2D) as well as differential messenger RNA expression (Figure 2E) between the corresponding inbred lines revealed a positive correlation between parental differences and heterosis, sRNA-based parental distances (Figure 2F) showed a negative correlation with MPH for GY. Thus, in contrast to SNP and mRNA expression differences, the greater the differences in sRNA expression between parental lines the lower the level of heterosis in their hybrids (Figures 2D–F). These results demonstrate inherent differences for the relationship between sRNAs and heterosis compared to the relationships between heterosis and genetic and gene expression data. The capability of all three data types to separate heterotic groups by PCA (Frisch et al., 2010; Frascaroli et al., 2013; Figure 2C) indicates that they commonly capture substantial information on the genetics of heterosis. Interestingly and importantly, the contrasting negative correlation with heterosis of parental sRNA expression variation (Figures 2D–F) demonstrates that the information content of sRNA data differs substantially and does not simply reflect genetic or gene expression variation.

### Association of parental sRNA expression variation with heterotic outcomes

Association studies employ panels of genetically diverse lines to identify trait-controlling genomic regions using factors such as variation in sequence (typically SNPs), and RNA expression (Harper et al., 2012). To identify and localize any sRNAs specifically contributing to heterosis in maize, we investigated the association between differences in sRNA expression between seedlings of the genetically diverse parental inbred lines and MPH for GY in the corresponding hybrids of our factorial (Figure 2A). The hybrids were grouped into two equally sized groups, by ranking them according their MPH for GY, resulting in a low and a high heterotic group for GY. Next, we tested the null hypothesis that differential sRNA expression occurs with the same probability in the parents of hybrids showing high or low heterosis by binomial testing. sRNAs whose expression patterns significantly reject the null hypothesis after false discovery rate (FDR) correction were called associated to high or low MPH for GY.

**Figure 3:**
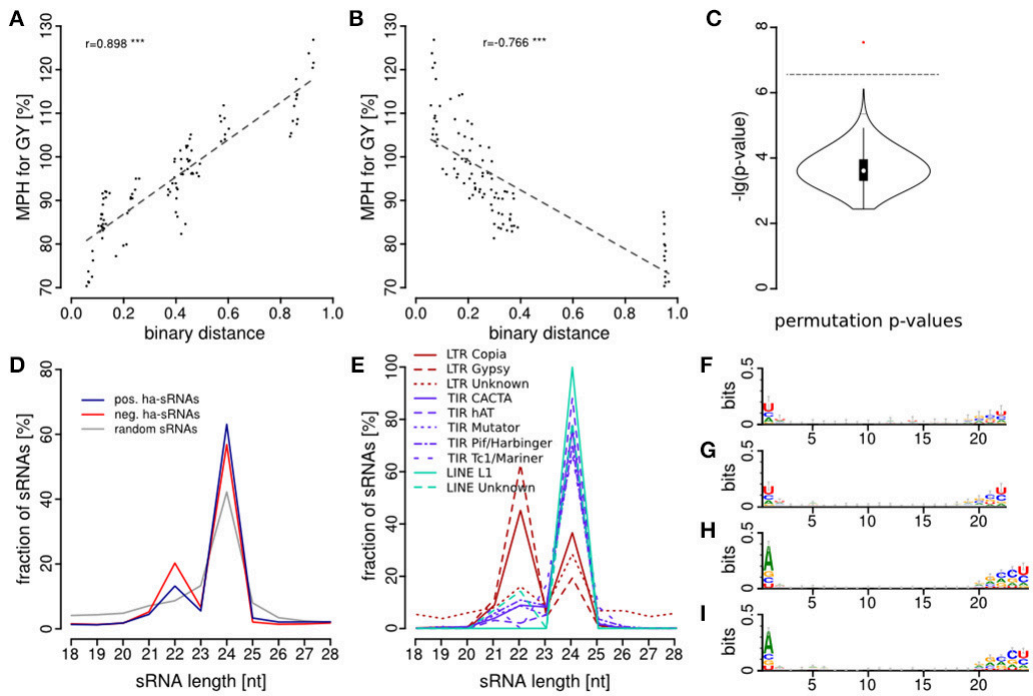
Identification and classification of heterosis-associated sRNAs. **(A,B)** Correlation of the number of differentially expressed ha-sRNAs between inbred parents (binary distance) with MPH for GY in the resulting hybrids with binary distances reveal strong correlations based on **(A)** 3,485 positively and **(B)** 6,282 negatively ha-sRNAs (^***^p < 0.001). **(C)** Permutation analysis with shuffled hybrid trait values. The lowest *p*-values of each permutation run (black violin plot) and of the actual genotype-trait allocation (red dot) are represented. The dotted line indicates the threshold to reach significance at 5% FDR. **(D)** Size distribution of positively/negatively ha-sRNAs and random sets of sRNAs; enrichment of 22 and 24-nt positively and negatively ha-sRNAs (bootstrap analysis, *p* < 0.001). **(E)** Mapping of ha-sRNAs to repeat super-families reveals distinct size distributions. See also Supplementary Figure 2 for statistics of enrichments and depletions. **(F–I)** Sequence motives of ha-sRNAs. **(F)** pos. 22-nt. **(G)** neg. 22-nt. **(H)** pos. 24-nt. **(I)** neg. 24-nt.

In total we identified 9,767 sRNAs, which showed an association of their differential expression between parental lines and MPH for GY of their hybrids. Of these 9,767 sRNAs, 3,485 showed an association with high mid-parental heterosis (MPH) for grain yield and 6,282 were associated with low MPH for grain yield (*p* < 0.05, FDR-adjusted binomial tests). We classified these sRNAs as positively or negatively heterosis-associated sRNAs (ha-sRNAs), respectively and use these specific terms in this manuscript. In agreement with the overall negative correlation of parental sRNA expression differences and heterosis (Figure 2F), the proportion of negatively ha-sRNA was higher with 64.3%. Three major canonical functional sRNA size classes, i.e., 21-, 22-, and 24-nt sRNAs, exhibit in each case a higher absolute number of negatively ha-sRNAs (Table 1). The correlations of the number of positively and negatively ha-sRNAs (integrated by binary distance) with MPH for GY show an overall steady increase and decrease (Figures 3A,B), respectively, which may indicate a linear, quantitative relationship between parental ha-sRNA expression variation and heterosis. This result is remarkable, since the binomial testing did not account for the actual value of MPH for GY within the two subclasses of hybrids with low or high GY. The binary distances of positively and negatively ha-sRNAs resulted in strong correlations of 0.89 and 0.76, respectively (Figures 3A,B). Separate analyses of the canonical size classes revealed similar correlation strength for each class (Supplementary Figure 1). Together these results indicate a strong general relationship between ha-sRNAs and heterosis for grain yield.

**Table 1:** Numbers and fractions of positively and negatively ha-sRNAs separately for canonical sRNA lengths.

**ha-sRNA length [nt]**	**pos. ha-sRNAs**	**neg. ha-sRNAs**
20	61 (36.53%)	106 (63.47%)
21	154 (32.08%)	326 (67.92%)
22	459 (26.52%) ±	1272 (73.48%)
24	2199 (38.11%)	3571 (61.89%)

To control for the possible explanation of random associations alone, we performed permutation tests, shuffling the hybrid trait values. These tests resulted in the loss of all associations—all 1000 permutations by far did not reach the significance level required to call any associated sRNA (Figure 3C). These results confirmed a statistically robust relationship between the specific parental sRNAs we identified and the phenotypic outcomes in F1 hybrids.

### Incidence of ha-sRNAs in B73 and Mo17 genotypes

To test whether the ha-sRNAs we identified are specific for the European inbred lines, we searched for identical sequences in the reference genotype B73 and the inbred line Mo17, which are commonly used for research on hybrids and heterosis. Based on the comprehensive dataset of Regulski et al. (2013) we identified 4808 ha-sRNAs in both genotypes, indicating a certain level of conservation of ha-sRNAs even between distant maize genotypes. The fractions of positively and negatively associated sRNAs (32.25 and 67.75%, respectively) among the conserved ha-sRNA set reflect the same pattern as the whole set of ha-sRNAs from European lines. Looking at sRNA of different lengths separately, more negatively ha-sRNAs of all lengths were expressed in B73 and Mo17 (Supplementary File S5).

### Cluster analysis confirms association of loci producing multiple sRNAs with heterotic phenotypes

In previous studies on maize sRNA expression, the analysis of sRNAs was based on the identification of clusters and repeat-normalization of expression levels. We used the maize B73 reference assembly and applied a strategy to identify sRNA clusters similar to the approach of Barber et al. (2012) (details in Methods) with at least 5 distinct sRNA within 200 bp defining a cluster and an expression threshold of at least 5 repeat-normalized rpmqn. Application of our association approach with MPH for GY to these clusters resulted in the identification of 47 and 194 positively and negatively heterosis associated-clusters, respectively (*p* < 0.05, FDR-adjusted binomial tests). These results resemble our initial associations of individual sRNAs with a larger fraction of 80.5% negatively associated clusters. The binary distances of positively and negatively associated clusters revealed a correlation of 0.87 and −0.62 with MPH for GY, respectively. Permutations of hybrid trait values again confirmed the statistical robustness. This alternative cluster-based analysis with repeat-normalized data confirmed the overall negative relation of parental sRNA expression variation with heterosis as well as the remarkable strength of the relations.

To facilitate the individual analysis of different sRNA lengths and to account for the assumption that every unique sRNA has the potential to act on co-opted or coincidental target sites in the genome or transcriptome that mediate gene regulatory properties, we continued our analysis with ha-sRNA, which we identified on the basis of individual sRNAs.

### Heterosis associated-sRNAs are enriched from specific genomic sequences

The expression levels of ha-sRNAs generally follow the abundance distribution of all sRNAs, but show an increased representation at 1–2 rpmqn and decreased representation of higher abundance classes (Supplementary File S6).

For more detailed characterization of the ha-sRNAs we tested for homology to rRNAs or tRNAs and found minor fractions of 1.3 or 0.1% of ha-sRNA overlapping with these sequence classes, respectively, which approximately equals the proportions of these sequence classes of the total sRNA population sequenced (Supplementary File S7). Among all known microRNAs in miRbase (Griffiths-Jones et al., 2006) only one positively ha-sRNA exhibits identity to zma-miR166g-5p. In summary, these analyses do not provide evidence to support strong, direct relations of parental expression variations with heterosis for sRNA sequences derived from annotated rRNA, tRNA or miRNA genes. This strongly non-random association, with the insignificant contribution of three major and abundant sRNA classes, further supports the validity of our permutation analysis.

Conversely, we discovered a strong enrichment of ha-sRNAs for sRNAs with lengths of 22- and 24-nt (*p* < 0.05, bootstrap analysis, Figure 3D). Mapping ha-sRNAs to annotated sequences of the maize genome revealed a strong enrichment of ha-sRNAs of both lengths for mapping to repeats. The different ha-sRNA classes show specific enrichment and depletion patterns for repeat super-families. While 22-nt ha-sRNAs are enriched for LTR Gypsy type TEs, 24-nt ha-sRNAs are enriched for various super-families of DNA TEs (*p* < 0.05, bootstrap analysis, Figure 3E and Supplementary Figure 1). Together, these findings suggest that short interfering RNAs (siRNAs), which act to silence transposable elements (TE) by RNA-directed DNA methylation (RdDM) (Slotkin and Martienssen, 2007), are important in regulating heterosis. The positive correlation between DNA methylation levels and levels of 22- and 24-nt sRNAs (He et al., 2013; Gent et al., 2014) as well as frequent loss and gain of DNA-methylation in hybrids (He et al., 2010; Chodavarapu et al., 2012; Greaves et al., 2012; Eichten et al., 2013) is consistent with this interpretation. Interestingly, mapping ha-sRNAs to TE exemplars, classified according to (Diez et al., 2014), revealed clear distinct trends: While 24-nt negatively ha-sRNAs map preferentially to DNA transposons, many of the 22-nt positively and especially negatively ha-sRNAs share sequence identity with long, high copy number retroelements that exhibit no evidence of recent insertions (Supplementary File S8).

### A 5′ nucleotide bias infers functionality of ha-sRNAs via different argonaute proteins

The identity of the 5′ nucleotide plays a key role in sorting of sRNAs into specifc Argonaute proteins in plants (Borges and Martienssen, 2015; Fang and Qi, 2016). We therefore analyzed the nucleotide composition of ha-sRNAs. While 22-nt ha-sRNAs showed a 5′ nucleotide bias for uridine and the least represented nucleotide was guanine at this position, the 24-nt ha-sRNAs exhibited a 5′ nucleotide bias for adenine and a nearly equal representation of the other three nucleotides (Figures 3F–I). Inferred from data in *Arabidopsis* the 5′ bias of 24-nt ha-sRNAs for adenine is in agreement with an involvement in RdDM, whereas the 5′ bias of 22-nt ha-sRNAs for uridine might indicate an involvement in post transcriptional gene silencing (Borges and Martienssen, 2015; Fang and Qi, 2016).

### ha-sRNAs in in-bred parents associate with differentially expressed transcripts

**Figure 4:**
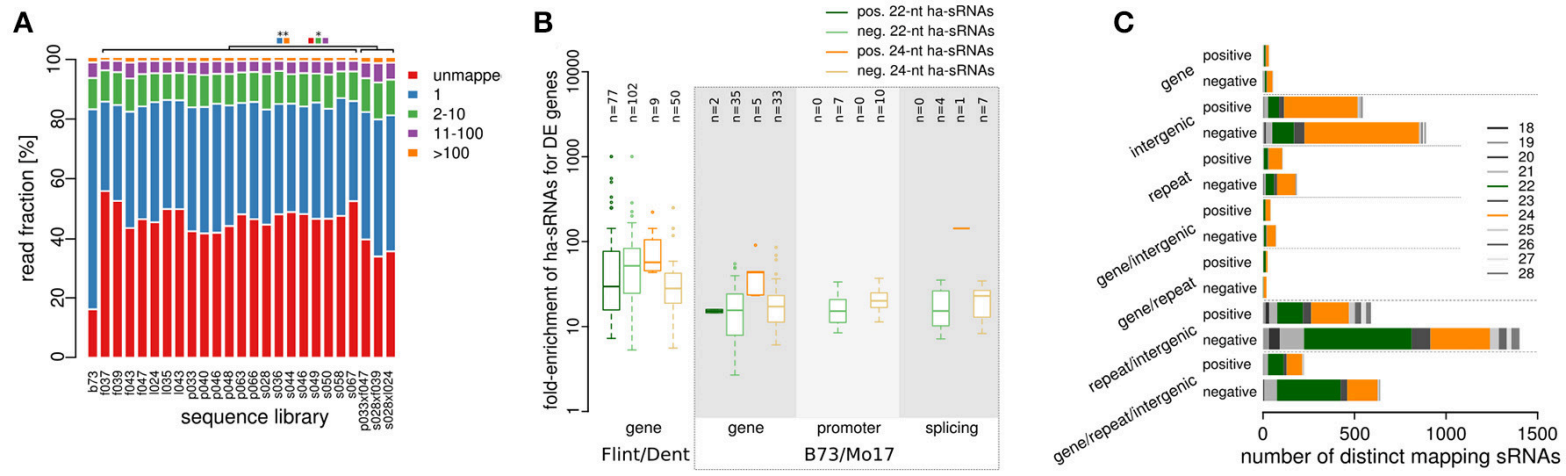
Relation of heterosis-associated sRNAs to genomic features and gene expression patterns. **(A)** sRNA mapping counts for all 21 inbred lines, 3 hybrids and the B73 reference sample. **(B)** Enrichment of 22-nt and 24-nt ha-sRNAs to loci of differentially expressed (DE) genes in the original European genotypes (Thiemann et al., 2010), and DE genes, alternative promoter use and alternative splicing in the genotype B73 and Mo17 (data set of Regulski et al., 2013). The mean enrichment factor of ha-sRNAs over the bootstrap results is plotted and the numbers of genes affected are shown above the boxplots. **(C)** Size distribution of ha-sRNAs mapping to single or multiple annotated features of the maize genome. Negatively 22-nt ha-sRNAs map primarily to multiple annotations (gene/intergenic, gene/repeat/intergenic), while 24-nt ha-sRNAs map primarily to single annotations (intergenic or repeat).

Transcriptional changes of protein-coding genes, which are held to largely underlie heterotic phenotypes (Chen, 2013), may be triggered by *trans* effects of ha-sRNAs either as a result of spreading of chromatin states to neighboring genes during suppression of TE activity (Hollister and Gaut, 2009; Hollister et al., 2011; Eichten et al., 2012), direct action on transposon-derived sequences co-opted as regulatory elements in paramutation-like processes (Erhard et al., 2013; Regulski et al., 2013), or coincidental partial complementarity to mRNAs inducing post transcriptional gene silencing (McCue et al., 2013). Provided that ha-sRNAs impact on transcriptional changes of protein-coding genes by any of these mechanisms, a spatial relation of these sRNA's mapping positions and protein-coding sequences should exist. Strand specificity with respect to the transcripts could point to the latter mechanism. Thus we tested whether the sRNAs, which we identified to be specifically associated with heterosis, map more frequently near or in differentially expressed protein coding genes than randomly selected sets of sRNAs of equal length and size. We concurrently analyzed whether the sRNAs map to the sense or antisense strand of the transcribed region of the genes. Importantly, although this analysis is based on the B73 reference genome, between 38 and 53% of small RNAs found in our panel of European inbred lines map to it (Figure 4A, Supplementary File S2) and thus provide an adequate basis to characterize the relation of ha-sRNAs to features of the maize genome.

To determine the potential influence of ha-sRNA on gene expression, we used previously published resources to identify genes that are differentially expressed between in-bred parental lines. We first collated all differentially expressed transcripts between at least one inbred line combination of our Flint/Dent factorial from microarray data (Thiemann et al., 2010) and between the inbred lines B73 and Mo17 using an extensive RNA-seq dataset of messenger RNA and sRNA from comparable plant tissue samples (Regulski et al., 2013). All the transcriptional variation types captured in the latter RNA-seq dataset have been related to sRNA expression and/or DNA methylation (Enke et al., 2011; Gent et al., 2013, 2014; Regulski et al., 2013), including differences in expression level, promoter switching and alternative splicing. Mapping sRNAs within 1 kb up- to 1 kb downstream of coding regions of these differentially expressed genes revealed both 22- and 24-nt ha-sRNAs to be enriched for all transcriptional variation types (*p* < 0.05, bootstrap analysis, Figure 4B). Our maize 22-nt ha-sRNAs are highly enriched for mapping to LTR Gypsy type transposons (Figure 2E, Supplementary Figure 1), which are by far the most abundantly expressed TE class in maize (Vicient, 2010). LTR Gypsy type transposons are also one of the most abundant class of TE in maize and largely equally distributed throughout the genome (Meyers et al., 2001; Mroczek and Dawe, 2003). These previous findings concerning the genomic distribution of LTR Gypsy sequences might lead to the expectation that mapping of 22-nt sRNA is biased away from genes. Therefore, our results that show enriched mapping of 22-nt ha-sRNAs to differentially expressed genes (Figure 4B) are rather intriguing. Interestingly, the mean number of 22-nt ha-sRNAs mapping to the differentially expressed genes was higher than of 24-nt ha-sRNAs. Taken together, these data suggest that these two sRNA classes may operate via different pathways.

Corroborative support for the potentially unequal modes of action of the two sRNA classes is provided by highly contrasting general mapping frequencies. Typically more than 100 genomic position counts were recorded for 22-nt ha-sRNAs, yet 0 or 1 position counts prevailing for 24-nt ha-sRNAs (Supplementary Figure 3). Strand-specific mapping of ha-sRNAs within the transcribed regions of the same differentially expressed genes revealed no clear trend for one strand for both 22- and 24-nt ha-sRNAs (Supplementary File S9), which is consistent with transcriptional gene silencing by RdDM. However, we do not exclude any other mechanism of sRNA action by individual ha-sRNAs.

To investigate the potential role for ha-sRNAs in regulating gene expression further, we explored their relationship to the annotated maize genome, subdivided into generally annotated features: (1) transcribed, protein coding sequences (gene); (2) TE or repeats (repeats); and (3) sequences without one of the previous annotations (intergenic). The preferential mapping of 24-nt ha-sRNAs to just single features indicates that they may have restricted spatial activity, primarily acting on specific loci at their site of origin and allelic loci, whereas the high proportion of 22-nt ha-sRNAs mapping to multiple features point to their potential of *trans*-regulatory action on functional genes distant from the site of origin (Figure 4C).

### Pericentromeric enrichment of 22-nt negatively heterosis associated-sRNAs

**Figure 5:**
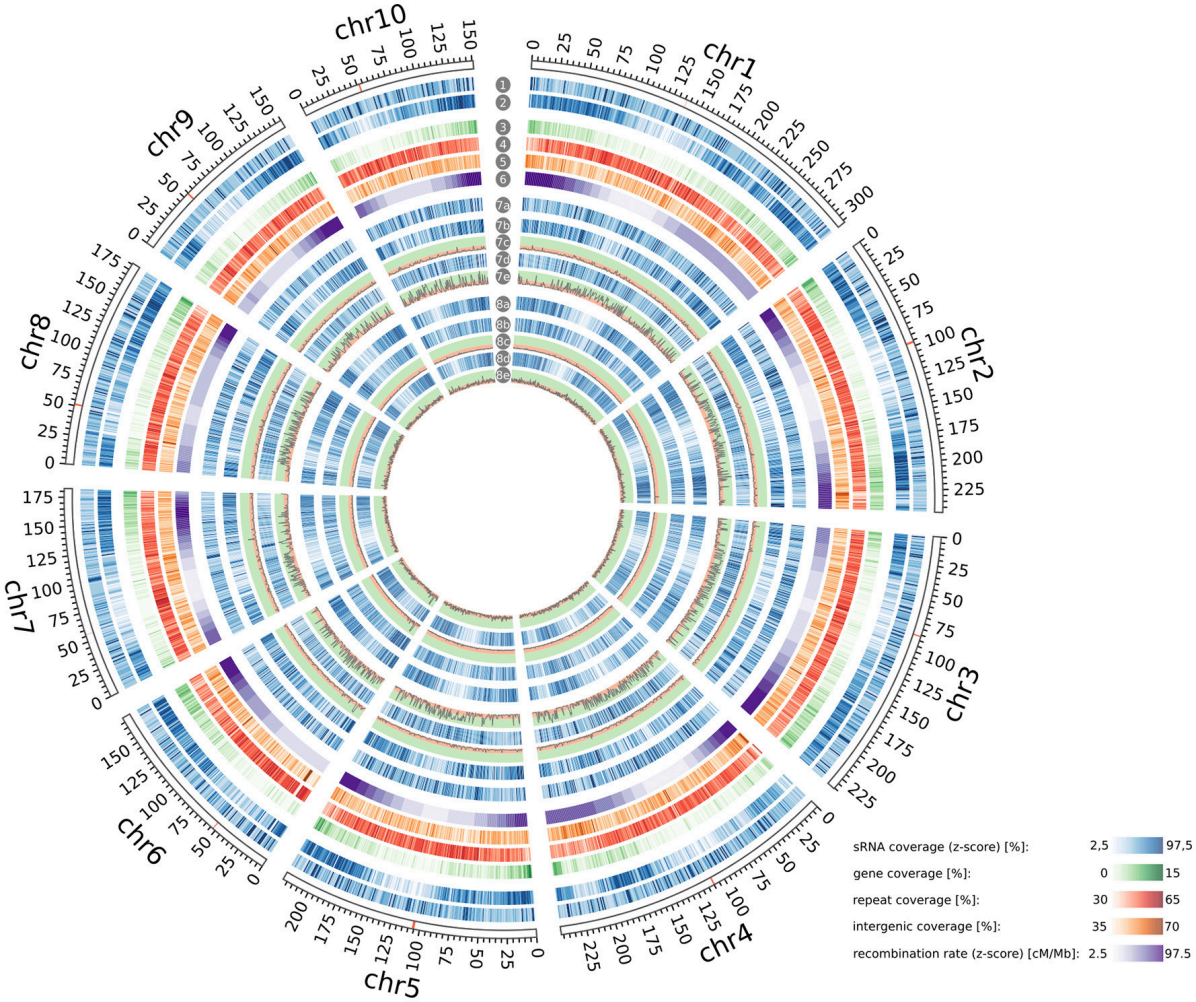
Genome-wide distribution and enrichment of sRNAs. Genomic coverage of ha-sRNAs (1), all sRNAs (2), genes (3), repeats (4), intergenic regions (5) and recombination rates (6) throughout the B73 reference genome. Distribution of 22-nt sRNAs (7a), positively 22-nt ha-sRNAs (7b), negatively 22-nt ha-sRNAs (7d), 24-nt sRNAs (8a), positively 24-nt ha-sRNAs (8b), negatively 24-nt ha-sRNAs (8d) on the B73 reference genome. −log_10_ plot of enrichment probabilities of positively 22-nt ha-sRNAs (7c), negatively 22-nt ha-sRNAs (7e), positively 24-nt ha-sRNAs (8c), and negatively 24-nt ha-sRNAs (8e). Peaks in green background zone show significant enrichment (*p* < 0.05). All distributions are shown in 1 Mb resolution. Centromeres according to Jiao et al. (2017) are indicated red in the rulers. Whole-genome visualization was created with Circos (Krzywinski et al., 2009). Annotations in (3–5) are according to genome assembly AGPv4.36.

To further characterize the ha-sRNAs we analyzed their genome-wide spatial distribution. Interestingly, ha-sRNAs are generally more evenly distributed than the whole sRNA population of the 21 inbred lines, with a better representation from the pericentromeric regions (Figure 5). To identify a potential causative fraction of ha-sRNAs for this difference, we tested for spatial enrichment of the 4 main subgroups of ha-sRNAs. To reiterate, these four groups were: (1) 22-nt with positive association to heterosis; (2) 22 nt with negative association to heterosis; (3) 24-nt with positive association to heterosis; and (4) 24-nt with negative association to heterosis. Unexpectedly, the 22-nt negatively ha-sRNAs are exclusively enriched in pericentromeric regions (*p* < 0.05, bootstrap analysis, Figure 5). Since this spatial distribution is closely correlated with recombination rate (Liu et al., 2009), having correlation coefficients ranging from −0.68 for chromosome 8 to −0.98 for chromosome 3 and 5 (Table 2), our inbred lines thus seem to contain largely “fixed” populations of 22-nt negatively ha-sRNAs. This spatial enrichment is interesting, since pericentromeric regions of the maize genome are thought to contribute disproportionally to heterosis, because of high residual heterozygosity resulting from strong recombinational suppression (Gore et al., 2009; McMullen et al., 2009). We found no such regional enrichment in any of the other ha-sRNA populations (i.e., 22-nt positively ha-sRNAs or both classes of 24-nt ha-sRNAs, *p* < 0.05, bootstrap analysis, Figure 5). In contrast, these three ha-sRNA populations showed a mainly positive correlation with recombination frequency (Table 2) and exhibited a more uniform distribution across all chromosomes, essentially following the gene/intergenic coverage and showing a clear, inverse relationship with the coverage of repeat sequences (Figure 5).

**Table 2:** Correlation of genomic ha-sRNA distributions with the recombination rate.

	**pos. 22-nt**	**neg. 22-nt**	**pos. 24-nt**	**neg. 24-nt**
chr1	0.952	−0.961	0.693	0.963
chr2	0.966	−0.955	0.945	0.962
chr3	0.925	−0.945	0.698	0.926
chr4	0.939	−0.897	0.948	0.771
chr5	0.899	−0.962	0.767	−0.141
chr6	0.417	−0.574	−0.516	0.756
chr7	0.914	−0.771	0.769	0.943
chr8	0.719	−0.696	0.617	0.513
chr9	0.810	−0.968	0.573	0.848
chr10	0.895	−0.809	0.754	−0.442

*Correlation coefficients of the different classes of ha-sRNAs for the 10 maize chromosomes*.

### Expression pattern of ha-sRNAs in hybrids and relation with heterotic outcomes

**Figure 6:**
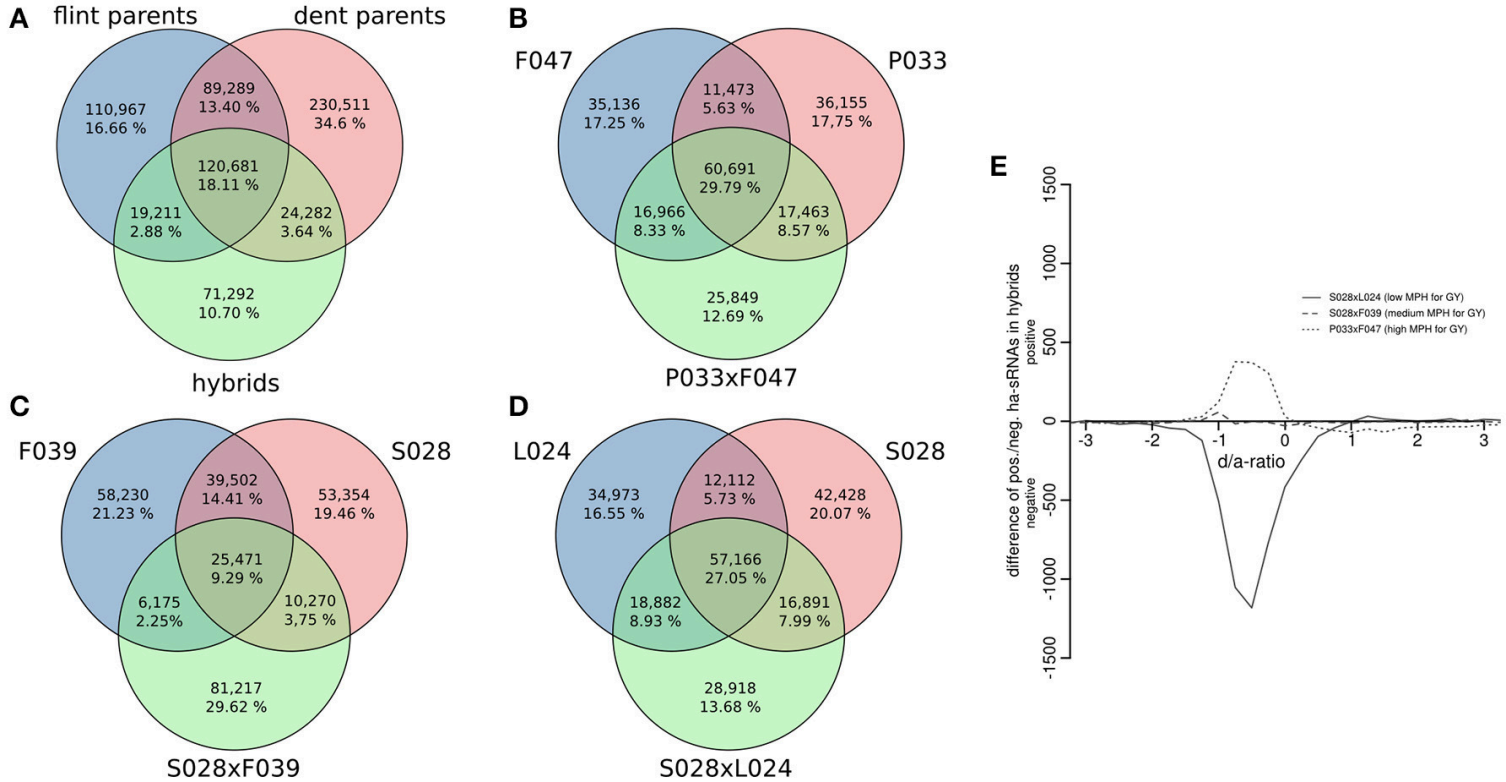
Expression pattern of sRNAs in hybrids. **(A)** Summarized numbers of specific and shared unique sRNAs for inbred lines of both heterotic groups and three hybrids. **(B–D)** sRNA population overlaps and specificities in individual triplets of parental inbred lines and the respective hybrid. **(E)** Hybrid dominance/additivity (d/a) expression pattern for hybrids with differing degrees of heterosis plotted as difference of positively and negatively ha-sRNAs. Overall, the expression pattern of ha-sRNAs tends toward the low-parent like expression. The subset size of prevailing positively/negatively ha-sRNAs resembles the degree of heterosis of the hybrid.

Expression pattern of sRNAs in hybrids are known to deviate from those of their parents, both with respect to sequence composition and expression levels (e.g., Groszmann et al., 2011; Barber et al., 2012; He et al., 2013). Therefore it follows that hybrid sRNA populations cannot be expected to constitute the sum of both parental populations with additive expression levels. Our analysis of 21 inbred and 3 hybrid genotypes confirmed that expression patterns of sRNAs in hybrids do indeed largely deviate from additive expression pattern. The mean numbers of specific and overlapping unique sRNAs for all Flint, Dent, and three hybrid genotypes analyzed at an expression threshold of 0.5 rpmqn are shown in Figure 6A and for each of the genotype triplets (the triplets consisting of the parental inbred lines and the respective hybrid) separately in Figures 6B–D. To determine the robustness of this pattern, the application of 10- or even 20-fold increased expression thresholds in the analysis did not change the general picture. Here, only the proportion of sRNAs specific to the three inbred parents pairs or hybrids increased slightly, while the overlapping fraction in all inbred and hybrid genotypes stayed below 35% (Supplementary File S10). This demonstrated that, irrespective of the expression level, many parental sRNAs are not necessarily expressed in the hybrids, and that hybrids express considerable numbers of sRNAs not present in the parents. Indeed, a significantly higher mapping rate for sRNAs of the hybrids from crosses of Flint and Dent inbred lines to the B73 reference genome indicated that more conserved genomic regions in hybrids produce sRNAs (Figure 3A, *p* < 0.05, FDR-adjusted two-sided heteroscedasdic *t*-test).

We then chose to analyse the actual pattern of ha-sRNA expression in hybrids. This was desirable and logical due to the well-founded trend for significantly non-additive sRNA expression in hybrids and, of course, since we originally identified the ha-sRNAs by associations of parental in-bred sRNA expression differences with MPH for GY of hybrids (rather than hybrid expression patterns). If the associations we have found between sRNA expression differences in parental in-breds and heterosis reflect some level of functional control, the numbers of negatively and positively ha-sRNAs should vary accordingly between hybrids with high or low heterosis. Consistent with this interpretation, we found the highest number of negatively ha-sRNAs to be expressed in a hybrid with low heterosis, and the highest number of positively ha-sRNAs to be expressed in a highly heterotic hybrid (Figure 6E). This independently derived relationship substantiates our claim that a multiplicity of functional sRNAs with antagonistic effects on heterosis has been identified.

## Discussion

### Association analysis of expression variation of sRNA in in-bred lines with MPH values in hybrids reveals a multiplicity of sRNAs, which are likely to function in heterosis

Heterosis refers to the phenomenon that first generation hybrids exhibit phenotypes superior either to the mean of the parents or to the better of the parents. Here, we provide unequivocal data that, for the first time, demonstrates that populations of sRNAs functionally contribute, in both a positive and negative manner, to the generation of this phenomenon. Furthermore, our analysis convincingly demonstrates that an important part of the molecular architecture of heterosis is built upon distinct sub-populations of sRNAs, potentially with different functional activities at the mechanistic level. Importantly, we identify a sub-population of sRNAs with unique features that we have dubbed 22-nt negatively heterosis-associated sRNAs. The predominance of negatively associated sRNA explains why there is a surprising overall negative correlation between parental in-bred line sRNA expression variation and heterosis.

By using MPH in our association approach to identify sRNAs specifically contributing to heterosis, we have uncovered the richness and complexity of sRNA populations involved in regulating heterotic outcomes. Despite the fact that all hybrids of our study show better-parent heterosis (BPH), we used MPH in our association approach. To search for factors of BPH is certainly interesting, since it is driving the exploitation of the phenomenon for agriculture of many species. However, naturally the values for BPH are smaller and thus result in less resolution and statistical power of association approaches. Tests we performed in advance to this end indicated that a sensible approach toward the role of sRNAs in BPH requires larger populations giving higher statistical power. From the genetic point of view, the increase over the parental mean constitutes the phenomenon heterosis and is a relevant measure to search for its molecular components (Kaeppler, 2012). Importantly, MPH describes heterosis in relation to both parents, which was appropriate for our searching for parental sRNA combinations that are related to the phenomenon.

Our approach revealed associations between two stages highly separated by developmental time. We used 7 day-old seedlings in our work for sRNA profiling, because they can be grown under highly controlled conditions to minimize environmental effects. The identification of highly significant associations between the early sRNA expression pattern of seedlings and the trait MPH of GY that take shape at the end of the plants life cycle, indicates robustness of our findings. Grain yield reflects “whole plant” performance and might well be reproduced by early sRNA expression pattern, since it is reliant on many aspects of plant growth and shows significant positive correlations with heterosis of many other traits (Flint-Garcia et al., 2009). Our approach to identify sRNAs contributing to heterosis is also distinct from previous work analyzing sRNA expression in maize in relation to gene expression regulation (e.g., Regulski et al., 2013; Gent et al., 2014; Madzima et al., 2014) or hybridization (Barber et al., 2012). This is because, intentionally, we did not filter or cluster the sRNAs beyond quantile and reads per million normalization to avoid any restriction and bias in identification of heterosis associated sRNAs. Indeed, the small numbers of heterosis associated-clusters we identified indicate that we would have lost many sRNAs by using this strategy exclusively, since clustering is dependent on mapping to the B73 reference assembly and the proportion of European line sRNAs mapping to B73 amounts merely approximately 50% (Figure 4A). We were conscious that the priori assumptions invoked in selective filtering of sequence datasets could prove restrictive in identification of sRNAs with effects on heterosis. Recent discoveries concerning the biogenesis of sRNAs—such as that Polymerase IV transcribes very short precursors, indicating single precursors for every siRNA (Zhai et al., 2015), as well as the identification of Dicer-independent small RNAs, which function as triggers for *de novo* DNA methylation (Ye et al., 2016)—provide justification for our approach to analyze individual sRNAs independently. Our approach also accounts for variation in the mode of action, given the possibility that *every* sRNA has the potential to mediate gene regulatory properties e.g., by acting on TE-derived co-opted regulatory elements (Erhard et al., 2013; Regulski et al., 2013) or through binding sites in the transcriptome that have arisen by coincidental complementarity (McCue et al., 2013). Finally, from the statistical point of view, filtering and thereby reducing the number of distinct sRNAs results in lower requirements for FDR correction and might have led to larger numbers of false positive associations. Thus when comparing our data with previous work it has to be taken into account that our method likely revealed higher numbers of individual sRNAs. Thus, whilst our data reveals a surprising number of sRNAs compared with similar work, this more inclusive analysis of genomic “dark matter” is expected to more fully represent the regulatory complexity at work in this phenomenon. By this approach in a population-level analysis, we uncovered new evidence pointing to hitherto hidden layers of regulatory complexity that will provide a unique foundation for a deeper understanding of this enigmatic phenomenon in the future.

### Sub-populations of ha-sRNAs may have different modes of biogenesis and activity

Whilst our study has uncovered an unexpected diversity of sRNAs influencing heterotic outcomes, bioinformatic analysis has also contributed some insights into their probable mechanisms of biogenesis and activity. Built on knowledge from the model plant *Arabidopsis* on the mode of biogenesis, 24-nt ha-sRNAs are likely to be derived from PolIV/PolV/RDR2 dependent silencing (Kim and Zilberman, 2015), especially as most are derived from intergenic and repeat regions (Figure 4C). In maize, a high abundance of 22-nt sRNAs has been found in the mediator of paramutation 1 mutant (MOP1), the homolog of RDR2, suggesting that their biogenesis is independent of MOP1 (Nobuta et al., 2008) and thus are likely to be generated by alternative pathways. In *Arabidopis* 22-nt sRNAs, mainly derived from microRNA precursors, have been described as triggers for secondary siRNA biogenesis, a feature that endows them with the properties of regulatory signal amplification and action in *trans* (Chen et al., 2010; Cuperus et al., 2010; Creasey et al., 2014). The high enrichment of 22-nt ha-sRNAs mapping to differentially expressed transcripts and their 5′ nucleotide bias for uridine is consistent with interactions with transcripts. Furthermore, secondary siRNAs that are active as post-transcriptional regulators of coding transcripts may also have a dual role as initiators of RdDM events (Wu et al., 2012). Interestingly, their lack of strand specificity and absence of any overlap with known microRNAs in miRbase (Griffiths-Jones et al., 2006), together with their mapping to differentially expressed transcripts, suggest that these 22-nt sRNAs populations may be enriched in secondary and even tertiary siRNAs, such as from phasiRNA cascades (Fei et al., 2013). Interestingly, the failure to identify substantial association between heterosis and microRNAs might represent late support to the suggestion by East in 1936 that heterosis is independent of developmental control (East, 1936), since many microRNAs are implicated in regulation of developmental switches (Chuck et al., 2009; Borges and Martienssen, 2015). However, we remain open to the possibility that highly conserved miRNAs with developmental roles may act as initiators of secondary siRNA biogenesis for 22-nt sRNAs identified in our study.

In summary, our data on the relation of ha-sRNAs with genes suggest that ha-sRNAs affect heterosis indirectly, either by targeting transcripts for PTGS or triggering secondary sRNAs and by targeting regulatory epigenetic marks to regions containing protein-coding genes that eventually result in gene expression changes across the genome with influence on the phenotype. Importantly, the fact that we can demonstrate this relationship for ha-sRNAs in both our European Flint/Dent factorial and in the distantly related B73/Mo17 genotype combination indicates a certain degree of conservation of ha-sRNA effects.

### Association studies provide unambiguous support for a role for sRNAs in restraining heterosis

While the strong and highly significant antagonistic correlations of ha-sRNAs suggest a general functional role of these sRNAs in heterosis, the higher number of negatively ha-sRNAs and the overall negative correlation of parental sRNA differences with MPH for GY indicate that the actions of sRNAs in hybrids add up to a negative contribution in heterosis formation. Following this assumption a general reduction of sRNAs should result in increased heterosis. In the *mop1* mutant of maize biogenesis of predominantly 24-nt sRNAs has been shown to be impeded (Nobuta et al., 2008) and the mutation was introgressed to the genotypes B73 and Mo17 to test for the effects on heterosis (Barber et al., 2012). The sRNA populations of these genotypes comprise a proportion of 67.75% negatively ha-sRNAs, the higher occurrence of this class mirroring that in the European lines. Considering 24-nt sRNA separately, the fraction of negatively ha-sRNAs was 59.55% in B73 and Mo17 genotypes (Supplementary File S5). The significant heterotic increase in developmental speed (days until 50% shed) and biomass (cob weight, stover biomass) reported in the maize mop1 mutant background (Barber et al., 2012) is essentially in agreement with a general reduction of 24-nt sRNAs. This overall reduction of this class consequently exerts a greater effect on a higher number of potentially negative-acting 24-nt ha-sRNAs, thus enhancing heterosis. These findings support our hypothesis on the functional role of sRNAs in heterosis, with the majority of sRNAs restraining heterosis.

An accumulated negative effect of sRNAs on heterosis is also consistent with Freeling's proposal (Freeling et al., 2012) that heterosis involves some deregulation of sRNA expression in hybrids. We can now add that there is a pattern in this deregulation. Certainly trans chromosomal methylation and demethylation (Greaves et al., 2012; Groszmann et al., 2013), where the methylation level of one parental allele changes to resemble that of the other parent, are involved and at least partly mediated by sRNAs. In the complex genome of maize these trans allelic methylation changes are in turn likely to trigger further substantial changes to the net sRNA expression pattern. The many newly expressed sRNAs in maize hybrids (Figures 6A–D) together with the combined set of two parental populations, might eventually lead to increased methylation that consequently lowers the gene expression potential of hybrids (Borges and Martienssen, 2015). Regulatory effects on gene expression by the transposon-silencing action of sRNAs are well documented (for review, see Volpe and Martienssen, 2011). Because the distribution and position of transposons in relation to genes determines the actual genes affected (Forestan et al., 2017), it is likely that the individual distribution of transposons in inbred lines has an influence on heterosis.

Interestingly, overall the expression of ha-sRNAs in hybrids is relatively low, being biased toward a point between mid- and low-parental levels (Figure 6E). Although the mechanism behind this finding is unclear, it is in agreement with other reports on relative sRNA expression levels in plant inbred lines and their hybrids (He et al., 2010; Groszmann et al., 2011; Barber et al., 2012; Shen et al., 2012), and may constitute a potential mechanism for counteracting an overall negative effect of sRNAs on heterosis. A lowered expression of sRNA in hybrids has been proposed to result in decreased TE silencing and the consequent upregulation of gene expression. This “transposon-gene expression tradeoff hypothesis” (Hollister et al., 2011) will be tested when more data on transcriptome size, which relates RNA level to gene content, in hybrids becomes available (Coate and Doyle, 2010). Equally, the fact that our data show a relatively low expression level of ha-sRNAs in post-germination development may constitute the residual “aftershock” situation of interactions that occur immediately post-fertilization when diverged genomes initially make contact. This would suggest that future work should focus on the establishment of heterotic interactions in early post-fertilization development in addition to post-germination development.

Nevertheless, the existence of negatively as well as positively ha-sRNAs and the prevalence of additive, mid-parental gene expression in hybrids (Swanson-Wagner et al., 2006; Thiemann et al., 2014), together with the occurrence of transcriptional complementation (Paschold et al., 2012) and examples of reduced gene expression associated with lowered methylation (Eichten et al., 2013) point to transcriptional regulation in hybrids being highly complex.

### A link between genomic architecture, recombination levels and ha-sRNAs regulating heterosis

**Figure 7:**
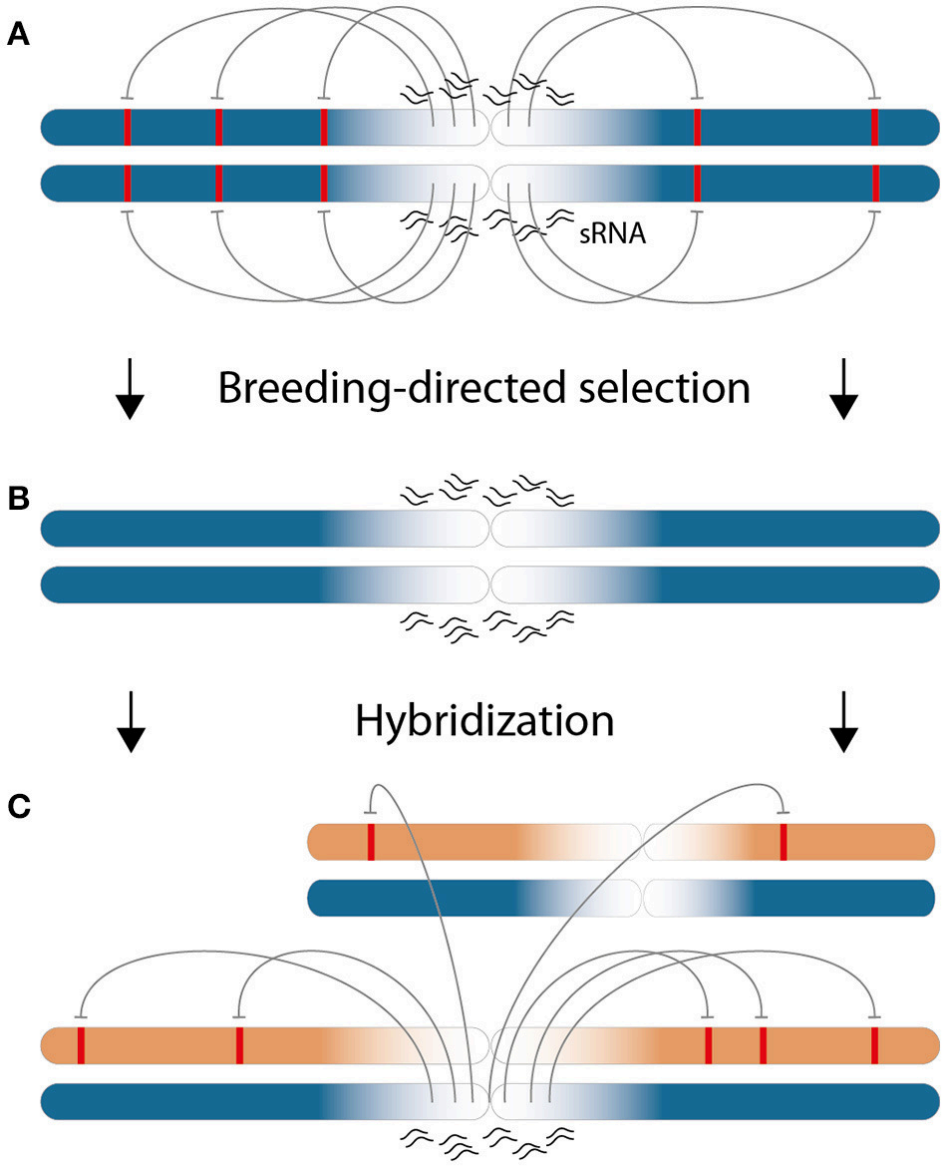
Model for the pericentromeric enrichment of negatively 22-nt ha-sRNAs by hybrid breeding. **(A)** Early in a hybrid breeding program, particular pericentromeric 22-nt sRNAs (wavy lines) exhibit gene regulatory effects in *trans* (arrows) on targets regions (red boxes) that are located on chromosome arms with negative effects on the phenotype. This situation is depicted for one parental chromosome pair of an inbred line of one heterotic group (blue). **(B)** Breeding to optimize inbred line phenotypes leads to the selection of alleles on chromosome arms without targets for the negative-acting 22-nt sRNAs. This is the only possibility against these negative regulatory effects, because of low recombination frequency (fading color of the chromosomes) the pericentromeric sRNA population do not change. Importantly, inbred line optimization occurs independently in two heterotic groups with different sRNA populations. **(C)** Thus, by hybridization, chromosomes from inbred lines of the other heterotic group (beige) with target regions (red boxes) come under the influence of negative-acting 22-nt sRNAs (arrows) from chromosomes of the first heterotic group (blue). This influence pertains the whole genome and retains heterosis according to the number of negative-acting 22-nt sRNAs that differ between the parental inbred lines.

It is interesting to ask why the specific subset of 22-nt sRNAs, for which the differential expression between parental lines is associated with low heterosis, is predominantly located in pericentromeric regions. Provided that these sRNAs exert actual negative effects on the phenotype, the selection for high-performing inbred plants within separated parental populations could explain this enrichment by taking the recombinational suppression in these regions into account. The low recombination combined with sequence complexity in this region would lead to different fixed sets of pericentromeric sRNAs - an interpretation which is supported by the comparatively low overlap of sRNAs between heterotic groups (Figure 2B). That most of the 22-nt ha-sRNAs origin from long-term inactive transposons (Diez et al., 2014, Supplementary File S8) opens the possibility that sequence complexity was created by accumulated random mutations that have had split by the assignment of individuals to specific heterotic groups. Negative regulatory effects on gene expression by the transposon-silencing action of sRNAs are well documented (for review, see Volpe and Martienssen, 2011). That the negative association of 22-nt sRNAs with GY heterosis coincides with a largely increased mapping frequency (please compare Supplementary Figures 3B,C) and also the predominant mapping to multiple genomic features (Figure 4C) strongly indicate that the action of 22-nt negatively ha-sRNAs reach beyond their sites of origin. Thus, we propose prevailing phenotypic depression by repeat-derived pericentromeric 22-nt sRNAs due to genome-wide negative effects on gene expression in *trans* at the beginning of a hybrid breeding program. Breeding-directed selection in the course of optimizing inbred plant performance would recombine chromosome arms carrying genes that are not targeted by the set of pericentromeric sRNAs with negative regulatory effects. Selection ideally ends up with optimized 22-nt sRNA-target combinations in inbred lines. Importantly, this optimization occurs in two separated heterotic groups and thus selection against negative regulatory effects of pericentromeric sRNAs on alleles of the other heterotic group is not possible. Therefore, we hypothesize that intergroup hybridizations result in *trans-*acting pericentromeric 22-nt sRNAs of one parent to exhibit negative regulatory effects on alleles of the other parent in hybrids, which are detrimental for the phenotype and lessen the heterotic response. The hypothetical model we propose is illustrated in Figure 7.

Whilst we are the first to describe this pericentromeric phenomenon in generating negative effects in hybrids, a previous study of the molecular basis of rare cases of hybrid inviability in specific crosses between in-breeding *Arabidopsis thaliana* ecotype lines implicated incompatibilities (hybrid necrosis) generated from combinations of rapidly evolving disease resistance loci that are otherwise harmless in their native genomic context (Bomblies et al., 2007). Thus, our data supports the notion that specific genomic regions may function disproportionately in generating negative effects in hybrid combinations.

### sRNAs as a dimension of epistatic interactions in heterosis

With respect to classical genetic models to explain heterosis (for review see Lippman and Zamir, 2007; Birchler et al., 2010), the mobility and *trans*-acting activity of sRNAs relate them intuitively to epistasis, which involve the interaction of distant alleles. In a theoretical framework of quantitative genetics epistatic interactions of individual loci with the entire genetic background were identified as a major component of MPH (Melchinger et al., 2007). The features of ha-sRNAs are to large extent compatible with driving factors of these epistatic interactions. In agreement with the prevailing negative associations of sRNAs with heterosis, a recent modeling of heterosis indicates epistasis as a major factor for the collapse of heterosis after prolonged separation of parental populations (Emmrich et al., 2015). The underlying mechanism requires beneficial mutations to accumulate for one parental population with negative effects on hybrid phenotypes—a situation, which resembles our model on the action of 22-nt negatively ha-sRNA (Figure 7).

## Conclusions

We report here cumulative evidence for sRNAs as one of the many components in heterosis formation in maize. This includes (i) the overall negative correlations between parental sRNA diversity and GY heterosis, (ii) prevailing highly significant associations of sRNAs with low GY heterosis, (iii) highly significant enrichment of ha-sRNAs for differentially expressed genes, and (iv) an exclusive enrichment of a specific, size class of negatively associated ha-sRNAs in recombination-suppressed regions of the genome. Taken together, these findings suggest that a part of sRNAs action in hybrids constitutes a heterosis-repressing system that has never been previously reported. Overcoming such a barrier has the potential to unlock even greater heterosis and substantially increase future crop yields. Importantly, our sRNA-based association strategy has the potential to provide markers that could be used during the optimization of parental inbred lines to select against sRNA-based suppression of heterosis in future hybrids. Furthermore, it may open the way to new hybrid breeding strategies addressing recombinational constraints of complex crop genomes by providing aid to defining heterotic groups.

## Author contributions

AM and TS devised the field experiments, adjusted the field data and provided plant material. SS devised and planned the sRNA-seq experiments and association studies. AT, SE, and DR conducted the sRNA-seq experiments. FS performed the bioinformatics and statistical analyses. MF contributed to statistical analyses. FS, AT, RG-D, HD, and SS interpreted all data and wrote the manuscript. All authors provided intellectual input and approved the final manuscript.

### Conflict of interest statement

The authors declare that the research was conducted in the absence of any commercial or financial relationships that could be construed as a potential conflict of interest.
