# Supplementary material for: Parental Expression Variation of Small RNAs Is Negatively Correlated with Grain Yield Heterosis in a Maize Breeding Population
Source: Front Plant Sci. 2018 Jan 30;9:13. doi: 10.3389/fpls.2018.00013 (PMC5797689; doi:10.3389/fpls.2018.00013)
Supplement: Supplementary file 1 [file Table1.PDF]

Supplementary Material

Parental expression variation of small RNAs is negatively correlated with grain yield heterosis in a maize breeding population

Felix Seifert, Alexander Thiemann, Robert Grant-Downton, Susanne Edelmann, Dominika Rybka, Tobias A. Schrag, Matthias Frisch, Hugh G. Dickinson, Albrecht E. Melchinger, and Stefan Scholten*

Correspondence: Corresponding Author: stefan.scholten@uni-hamburg.de

Supplemetary Table

Supplementary File S1 | Heterosis values of the hybrids

Values for mid-parental heterosis (MPH) for grain yield (GY) in % are given.

Line	F037	F039	F043	F047	L024	L035	L043
P033	101.45	108.1	99.18	123.73	86.3	98.96	108.85
P040	90.72	95.99	87.73	111.65	77.24	87.09	97.21
P046	104.10	109.38	102.52	126.81	87.3	102.14	111.78
P048	98.41	104.01	95.54	121.53	84.59	96.31	106.43
P063	91.47	97.29	90.48	113.26	79.66	90.38	99.48
P066	96.11	103.54	96.29	120.4	82.35	96.13	105.14
S028	84.60	93.93	84.57	105.4	72.51	84.14	90.48
S036	89.05	95.27	86.17	108.62	73.73	86.84	96.05
S044	95	102.52	92.46	117.82	79.92	94.99	105.10
S046	91.68	98.88	91.96	114.33	76.25	90.42	99.45
S049	83.77	92.54	85.79	108.21	70.38	84.20	93.92
S050	83.57	91.95	85.50	107.52	71.22	83.83	92.56
S058	81.34	91.24	83.14	105.66	71.37	82.90	92.90
S067	90.69	99.54	92.08	114.06	78.41	91.60	101.45
