# Supplementary material for: Parental Expression Variation of Small RNAs Is Negatively Correlated with Grain Yield Heterosis in a Maize Breeding Population
Source: Front Plant Sci. 2018 Jan 30;9:13. doi: 10.3389/fpls.2018.00013 (PMC5797689; doi:10.3389/fpls.2018.00013)
Supplement: Supplementary file 2 [file Table2.PDF]

Supplementary Material

Parental expression variation of small RNAs is negatively correlated with grain yield heterosis in a maize breeding population

Felix Seifert, Alexander Thiemann, Robert Grant-Downton, Susanne Edelmann, Dominika Rybka, Tobias A. Schrag, Matthias Frisch, Hugh G. Dickinson, Albrecht E. Melchinger, and Stefan Scholten*

Correspondence: Corresponding Author: stefan.scholten@uni-hamburg.de

Supplementary Table 2

Supplementary File S2 | Summary of sequencing data

sample	number of raw reads	number of processed reads	number of distinct processed reads	fraction of distinct reads mapping to B73 Refgen_v4 [%]
**F037**	22653720	19038867	1396788	38,57
**F039**	12848402	9703097	1470746	41,51
**F043**	21939051	14488783	4072399	45,13
**F047**	15269024	12681993	2557259	48,94
**L024**	24442183	19794581	2970112	49,52
**L035**	37307793	31142343	4286727	45,95
**L043**	32582844	24682433	3600852	45,13
**P033**	17119925	12052946	2217321	51,61
**P040**	18267239	14513727	2794270	53,35
**P046**	20963127	17371388	3184178	52,84

**P048**	13285411	11123942	2276086	51,11
**P063**	14112422	9145421	1826393	46,39
**P066**	12953371	11184753	2423948	48,84
**S028**	14132611	10466762	1675266	48,99
**S036**	18225524	12813503	1605377	45,31
**S044**	19053823	16066868	2212081	46,13
**S046**	14581363	12956834	1340702	46,56
**S049**	21233470	16805847	2872341	48,47
**S050**	21610221	11880077	1537121	47,49
**S058**	35305865	31084539	3139157	47,94
**S067**	18969844	12511557	1685637	41,76
**21 inbred lines**	426857233	331510261	31681594	40,83
**P033xF047**	35266505	23111082	4262626	48,80
**S028xF039**	13702409	8953310	2805689	50,62
**S028xL024**	28534979	17360183	3541357	52,45
**3 hybrids**	77503893	49424575	9385332	48,71
**B73**	16103827	12071379	2606781	77,64
**all libraries (21 inbred lines. B73. 3 hybrids)**	504361126	380934836	39104679	43,56
