# Supplementary material for: Parental Expression Variation of Small RNAs Is Negatively Correlated with Grain Yield Heterosis in a Maize Breeding Population
Source: Front Plant Sci. 2018 Jan 30;9:13. doi: 10.3389/fpls.2018.00013 (PMC5797689; doi:10.3389/fpls.2018.00013)
Supplement: Supplementary file 3 [file Table3.PDF]

# *Supplementary Material*

# Parental expression variation of small RNAs is negatively correlated with grain yield heterosis in a maize breeding population

**Felix Seifert, Alexander Thiemann, Robert Grant-Downton, Susanne Edelmann, Dominika Rybka, Tobias A. Schrag, Matthias Frisch, Hugh G. Dickinson, Albrecht E. Melchinger, and Stefan Scholten***

**Correspondence:** Corresponding Author: stefan.scholten@uni-hamburg.de

**Supplementary Table 3**

**Supplementary File S3 | Overlaps of sRNAs between the parental inbred lines of the 98 hybrids**

| flint line | dent line | number in both lines | flint | both | dent |
|---|---|---|---|---|---|
| f037 | p033 | 194517 | 35.336 | 31.324 | 33.340 |
| f037 | p040 | 194813 | 36.794 | 29.764 | 33.441 |
| f037 | p046 | 194128 | 36.596 | 30.198 | 33.206 |
| f037 | p048 | 195241 | 35.576 | 30.837 | 33.587 |
| f037 | p063 | 196238 | 35.126 | 30.950 | 33.925 |
| f037 | p066 | 194009 | 35.811 | 31.024 | 33.165 |
| f037 | s028 | 197650 | 34.937 | 30.666 | 34.397 |
| f037 | s036 | 199174 | 35.192 | 29.909 | 34.899 |
| f037 | s044 | 193347 | 34.945 | 32.118 | 32.937 |
| f037 | s046 | 203294 | 36.115 | 27.667 | 36.218 |
| f037 | s049 | 189807 | 35.127 | 33.187 | 31.686 |

| flint line | dent line | number in both lines | flint | both | dent |
|---|---|---|---|---|---|
| f037 | s050 | 197553 | 34.512 | 31.123 | 34.364 |
| f037 | s058 | 188817 | 34.813 | 33.860 | 31.328 |
| f037 | s067 | 197402 | 34.855 | 30.830 | 34.314 |
| f039 | p033 | 191663 | 34.373 | 33.130 | 32.497 |
| f039 | p040 | 189220 | 34.926 | 33.448 | 31.626 |
| f039 | p046 | 190473 | 35.379 | 32.545 | 32.075 |
| f039 | p048 | 190995 | 34.144 | 33.595 | 32.261 |
| f039 | p063 | 194615 | 34.585 | 31.894 | 33.521 |
| f039 | p066 | 187378 | 33.539 | 35.507 | 30.953 |
| f039 | s028 | 193002 | 33.370 | 33.664 | 32.965 |
| f039 | s036 | 193951 | 33.447 | 33.259 | 33.293 |
| f039 | s044 | 191952 | 34.472 | 32.929 | 32.599 |
| f039 | s046 | 199443 | 34.882 | 29.988 | 35.130 |
| f039 | s049 | 184627 | 33.307 | 36.768 | 29.925 |
| f039 | s050 | 194668 | 33.542 | 32.919 | 33.539 |
| f039 | s058 | 184470 | 33.276 | 36.859 | 29.865 |
| f039 | s067 | 193867 | 33.667 | 33.068 | 33.265 |
| f043 | p033 | 181402 | 30.661 | 36.024 | 33.314 |
| f043 | p040 | 178893 | 31.169 | 36.451 | 32.379 |

| flint line | dent line | number in both lines | flint | both | dent |
|---|---|---|---|---|---|
| f043 | p046 | 178758 | 31.144 | 36.528 | 32.328 |
| f043 | p048 | 183409 | 31.420 | 34.536 | 34.044 |
| f043 | p063 | 187245 | 32.010 | 32.595 | 35.395 |
| f043 | p066 | 182325 | 31.697 | 34.651 | 33.652 |
| f043 | s028 | 184920 | 30.458 | 34.959 | 34.583 |
| f043 | s036 | 191238 | 32.503 | 30.753 | 36.744 |
| f043 | s044 | 188922 | 33.421 | 30.610 | 35.969 |
| f043 | s046 | 199316 | 34.840 | 25.852 | 39.308 |
| f043 | s049 | 180952 | 31.953 | 34.899 | 33.149 |
| f043 | s050 | 193365 | 33.094 | 29.466 | 37.440 |
| f043 | s058 | 179584 | 31.461 | 35.900 | 32.639 |
| f043 | s067 | 193510 | 33.545 | 28.968 | 37.487 |
| f047 | p033 | 177884 | 29.290 | 40.568 | 30.142 |
| f047 | p040 | 173271 | 28.936 | 42.782 | 28.282 |
| f047 | p046 | 174617 | 29.511 | 41.653 | 28.835 |
| f047 | p048 | 178627 | 29.584 | 39.983 | 30.433 |
| f047 | p063 | 185038 | 31.199 | 35.958 | 32.843 |
| f047 | p066 | 174140 | 28.487 | 42.873 | 28.640 |
| f047 | s028 | 178981 | 28.150 | 41.279 | 30.570 |
| f047 | s036 | 186243 | 30.693 | 36.030 | 33.277 |

| flint line | dent line | number in both lines | flint | both | dent |
|---|---|---|---|---|---|
| f047 | s044 | 185322 | 32.128 | 34.926 | 32.946 |
| f047 | s046 | 193842 | 33.000 | 31.107 | 35.893 |
| f047 | s049 | 173297 | 28.947 | 42.760 | 28.293 |
| f047 | s050 | 188553 | 31.386 | 34.519 | 34.095 |
| f047 | s058 | 171231 | 28.118 | 44.455 | 27.428 |
| f047 | s067 | 190036 | 32.330 | 33.061 | 34.609 |
| l024 | p033 | 178270 | 29.443 | 39.628 | 30.929 |
| l024 | p040 | 175000 | 29.638 | 40.723 | 29.638 |
| l024 | p046 | 175652 | 29.927 | 40.178 | 29.899 |
| l024 | p048 | 178649 | 29.593 | 39.332 | 31.075 |
| l024 | p063 | 185820 | 31.489 | 34.776 | 33.735 |
| l024 | p066 | 175958 | 29.226 | 40.753 | 30.021 |
| l024 | s028 | 182452 | 29.517 | 37.971 | 32.512 |
| l024 | s036 | 186503 | 30.789 | 35.233 | 33.978 |
| l024 | s044 | 185050 | 32.028 | 34.512 | 33.460 |
| l024 | s046 | 193008 | 32.711 | 31.086 | 36.203 |
| l024 | s049 | 174748 | 29.537 | 40.926 | 29.537 |
| l024 | s050 | 188875 | 31.503 | 33.689 | 34.807 |
| l024 | s058 | 175931 | 30.038 | 39.951 | 30.011 |

| flint line | dent line | number in both lines | flint | both | dent |
|---|---|---|---|---|---|
| I024 | s067 | 189711 | 32.214 | 32.691 | 35.094 |
| I035 | p033 | 177551 | 29.157 | 38.975 | 31.868 |
| I035 | p040 | 175902 | 29.999 | 38.772 | 31.229 |
| I035 | p046 | 173868 | 29.208 | 40.367 | 30.425 |
| I035 | p048 | 177357 | 29.080 | 39.127 | 31.795 |
| I035 | p063 | 181549 | 29.877 | 36.755 | 33.368 |
| I035 | p066 | 177559 | 29.864 | 38.265 | 31.871 |
| I035 | s028 | 183046 | 29.746 | 36.341 | 33.913 |
| I035 | s036 | 188567 | 31.547 | 32.605 | 35.848 |
| I035 | s044 | 181472 | 30.688 | 35.972 | 33.340 |
| I035 | s046 | 193970 | 33.044 | 29.321 | 37.635 |
| I035 | s049 | 175468 | 29.826 | 39.115 | 31.059 |
| I035 | s050 | 188146 | 31.238 | 33.057 | 35.705 |
| I035 | s058 | 174868 | 29.613 | 39.565 | 30.823 |
| I035 | s067 | 189027 | 31.969 | 32.027 | 36.004 |
| I043 | p033 | 176071 | 28.562 | 40.647 | 30.791 |
| I043 | p040 | 173761 | 29.137 | 40.993 | 29.871 |
| I043 | p046 | 173941 | 29.238 | 40.819 | 29.943 |
| I043 | p048 | 175894 | 28.490 | 40.789 | 30.721 |
| I043 | p063 | 180773 | 29.576 | 37.833 | 32.591 |

| flint line | dent line | number in both lines | flint | both | dent |
|---|---|---|---|---|---|
| I043 | p066 | 173652 | 28.286 | 41.887 | 29.827 |
| I043 | s028 | 179550 | 28.378 | 39.490 | 32.132 |
| I043 | s036 | 183679 | 29.725 | 36.617 | 33.658 |
| I043 | s044 | 179611 | 29.970 | 37.875 | 32.155 |
| I043 | s046 | 189874 | 31.600 | 32.578 | 35.822 |
| I043 | s049 | 170055 | 27.592 | 44.065 | 28.343 |
| I043 | s050 | 184503 | 29.880 | 36.166 | 33.954 |
| I043 | s058 | 171894 | 28.395 | 42.496 | 29.109 |
| I043 | s067 | 184079 | 30.140 | 36.058 | 33.802 |
| all flint* | all dent* | 594941 | 21.881 | 35.293 | 42.827 |

* corrected for sRNAs in opposing heterotic group in any other parental combination.
