# Supplementary material for: Parental Expression Variation of Small RNAs Is Negatively Correlated with Grain Yield Heterosis in a Maize Breeding Population
Source: Front Plant Sci. 2018 Jan 30;9:13. doi: 10.3389/fpls.2018.00013 (PMC5797689; doi:10.3389/fpls.2018.00013)
Supplement: Supplementary file 4 [file Table4.PDF]

Supplementary Material

Parental expression variation of small RNAs is negatively correlated with grain yield heterosis in a maize breeding population

Felix Seifert, Alexander Thiemann, Robert Grant-Downton, Susanne Edelmann, Dominika Rybka, Tobias A. Schrag, Matthias Frisch, Hugh G. Dickinson, Albrecht E. Melchinger, and Stefan Scholten*

Correspondence: Corresponding Author: stefan.scholten@uni-hamburg.de

Supplementary Table 4

Supplementary File S4 | Overlap of sRNAs between heterotic groups at various expression thresholds

expression threshold [rpmqn]		0.5	1	2	5	10
# of distinct sRNAs	flint/dent	594941	227795	103699	40404	20422
fraction of sRNAs [%]	flint	21.88	19.52	17.82	16.09	15.04
	dent	42.83	35.41	31.37	28.39	27.06
	flint/dent	35.29	45.07	50.81	55.52	57.89
