# Supplementary material for: Parental Expression Variation of Small RNAs Is Negatively Correlated with Grain Yield Heterosis in a Maize Breeding Population
Source: Front Plant Sci. 2018 Jan 30;9:13. doi: 10.3389/fpls.2018.00013 (PMC5797689; doi:10.3389/fpls.2018.00013)
Supplement: Supplementary file 5 [file Table5.PDF]

Supplementary Material

Parental expression variation of small RNAs is negatively correlated with grain yield heterosis in a maize breeding population

Felix Seifert, Alexander Thiemann, Robert Grant-Downton, Susanne Edelmann, Dominika Rybka, Tobias A. Schrag, Matthias Frisch, Hugh G. Dickinson, Albrecht E. Melchinger, and Stefan Scholten*

Correspondence: Corresponding Author: stefan.scholten@uni-hamburg.de

Supplementary Table 5

Supplementary File S5 | Incidence of ha-sRNAs in B73/Mo17 genotypes

**sRNA length**	**18**	**19**	**20**	**21**	**22**	**23**	**24**	**25**	**26**	**27**	**28**
# of sRNAs identified / fraction as to all (%)	81 / 1,68	98 / 2,04	106 / 2,20	299 / 6,22	1327 / 27,60	277 / 5,76	2111 / 43,91	129 / 2,68	107 / 2,23	134 / 2,79	139 / 2,89
# positively associated / fraction as to length subset (%)	23 / 28,40	26 / 26,53	36 / 33,96	72 / 24,08	284 / 21,40	93 / 33,57	854 / 40,45	56 / 43,41	38 / 35,51	48 / 35,82	44 / 31,65
# negatively associated / fraction as to length subset (%)	58 / 71,60	72 / 73,47	70 / 66,04	227 / 75,92	1043 / 78,60	184 / 66,43	1257 / 59,55	73 / 56,59	69 / 64,49	86 / 64,18	95 / 68,35
