# Supplementary material for: Parental Expression Variation of Small RNAs Is Negatively Correlated with Grain Yield Heterosis in a Maize Breeding Population
Source: Front Plant Sci. 2018 Jan 30;9:13. doi: 10.3389/fpls.2018.00013 (PMC5797689; doi:10.3389/fpls.2018.00013)
Supplement: Supplementary file 6 [file Table6.PDF]

# *Supplementary Material*

# Parental expression variation of small RNAs is negatively correlated with grain yield heterosis in a maize breeding population

**Felix Seifert, Alexander Thiemann, Robert Grant-Downton, Susanne Edelmann, Dominika Rybka, Tobias A. Schrag, Matthias Frisch, Hugh G. Dickinson, Albrecht E. Melchinger, and Stefan Scholten***

**Correspondence:** Corresponding Author: stefan.scholten@uni-hamburg.de

**Supplementary Table 6**

**Supplementary File S6 | Abundance distribution of sRNAs**

| abundance class [rpmqn] | all sRNA [%] | pos. ha-sRNA [%] | neg. ha-sRNA [%] |
|---|---|---|---|
| 0.5-1 | 44.63 | 43.18 | 35.30 |
| 1-2 | 24.78 | 41.46 | 45.25 |
| 2-5 | 16.71 | 11.76 | 13.05 |
| 5-10 | 6.43 | 2.52 | 3.51 |
| 10-20 | 3.46 | 0.68 | 1.32 |
| 20-50 | 2.31 | 0.22 | 0.97 |
| 50-100 | 0.80 | 0.05 | 0.33 |
| >100 | 0.84 | 0.08 | 0.23 |
