# Supplementary material for: Parental Expression Variation of Small RNAs Is Negatively Correlated with Grain Yield Heterosis in a Maize Breeding Population
Source: Front Plant Sci. 2018 Jan 30;9:13. doi: 10.3389/fpls.2018.00013 (PMC5797689; doi:10.3389/fpls.2018.00013)
Supplement: Supplementary file 7 [file Table7.PDF]

Supplementary Material

Parental expression variation of small RNAs is negatively correlated with grain yield heterosis in a maize breeding population

Felix Seifert, Alexander Thiemann, Robert Grant-Downton, Susanne Edelmann, Dominika Rybka, Tobias A. Schrag, Matthias Frisch, Hugh G. Dickinson, Albrecht E. Melchinger, and Stefan Scholten

Correspondence: stefan.scholten@uni-hamburg.de

Supplementary Table 7

Supplementary File S7 | Number and percentage of sRNAs with homology to known miRNA, tRNA and rRNA among all sRNAs sequenced and ha-sRNAs.

	**sRNA length**	**all**	**20-nt**	**21-nt**	**22-nt**	24-nt
**miRNA**	all sRNAs	134 (0.0004%)	16	93	24	0
	pos. ha-sRNAs	1 (0.0287%)	0	1	0	0
	neg. ha-sRNAs	0 (0.000%)	0	0	0	0
**rRNA SSU**	all sRNAs	73664 (0.2325%)	4089	4739	5036	6852
	pos. ha-sRNAs	55 (1.5782%)	4	12	7	4
	neg. ha-sRNAs	69 (1.0984%)	9	7	7	4
**rRNA LSU**	all sRNAs	80331 (0.2536%)	4580	5052	5333	5941
	pos. ha-sRNAs	4 (0.1148%)	0	0	1	2
	neg. ha-sRNAs	47 (0.7482%)	3	7	12	8
**tRNA**	all sRNAs	30209 (0.0954%)	1805	1803	1746	1928
	pos. ha-sRNAs	5 (0.1435%)	1	0	1	1
	neg. ha-sRNAs	4 (0.0637%)	0	1	0	1
