# Supplementary material for: Parental Expression Variation of Small RNAs Is Negatively Correlated with Grain Yield Heterosis in a Maize Breeding Population
Source: Front Plant Sci. 2018 Jan 30;9:13. doi: 10.3389/fpls.2018.00013 (PMC5797689; doi:10.3389/fpls.2018.00013)
Supplement: Supplementary file 8 [file Table8.PDF]

Supplementary Material

Parental expression variation of small RNAs is negatively correlated with grain yield heterosis in a maize breeding population

Felix Seifert, Alexander Thiemann, Robert Grant-Downton, Susanne Edelmann, Dominika Rybka, Tobias A. Schrag, Matthias Frisch, Hugh G. Dickinson, Albrecht E. Melchinger, and Stefan Scholten*

Correspondence: Corresponding Author: stefan.scholten@uni-hamburg.de

Supplementary Table 8

Supplementary File S8 | Enrichment of ha-sRNAs for mapping to transposable element exemplars classified according to Dietz et al., (2014) (p<0.05, bootstrap analysis)**.**

Exemplar class	# of TEs with positively ha-sRNAs	Enrichment factor* 22-nt / 24-nt		# of TEs with negatively ha-sRNAs	Enrichment factor* 22-nt / 24-nt	
	22-nt / 24-nt	mean	max	22-nt / 24-nt	mean	max
D	1 / 0	2 / -	2 / -	8 / 42	3.8 / 1.9	11.0 / 5.0
R1	0 / 0	- / -	- / -	0 / 7	- / 1.4	- / 3.0
R2	15 / 5	5.3 / 2.4	18.0 / 3	33 / 4	12.1 / 7.0	136.1 / 16.0
No class	6 / 39	2.7 / 1.4	6.0 /5.0	5 / 13	3.0 / 3.8	3.0 / 5.0

* Based on exemplars with a minimum of 2 mapped ha-sRNAs.
