# Supplementary material for: Parental Expression Variation of Small RNAs Is Negatively Correlated with Grain Yield Heterosis in a Maize Breeding Population
Source: Front Plant Sci. 2018 Jan 30;9:13. doi: 10.3389/fpls.2018.00013 (PMC5797689; doi:10.3389/fpls.2018.00013)
Supplement: Supplementary file 9 [file Table9.PDF]

Supplementary Material

Parental expression variation of small RNAs is negatively correlated with grain yield heterosis in a maize breeding population

Felix Seifert, Alexander Thiemann, Robert Grant-Downton, Susanne Edelmann, Dominika Rybka, Tobias A. Schrag, Matthias Frisch, Hugh G. Dickinson, Albrecht E. Melchinger, and Stefan Scholten*

Correspondence: Corresponding Author: stefan.scholten@uni-hamburg.de

Supplementary Table 9

Supplementary File S9 | Strand-specific mapping of ha-sRNAs to differentially expressed genes and enrichment analysis

		sequence length [nt]										
**sRNA class**		**18**	**19**	**20**	**21**	**22**	**23**	**24**	**25**	**26**	**27**	**28**
pos. ha-sRNAs. sense	counts	2	0	2	50	151	14	118	9	0	0	0
	enrichment	0.3	0.0	0.2	1.9	1.6	0.9	1.3	1.0	0.0	0.0	0.0
	p-values*	0.797	1	0.918	0.051	**0.021**	0.472	0.074	0.451	1	1	1
neg ha-sRNAs. sense	counts	2	1	12	233	890	44	299	9	0	0	0
	enrichment	0.1	0.1	0.6	3.7	3.0	1.3	2.1	0.8	0.0	0.0	0.0
	p-values*	1	0.996	0.808	**0.001**	**0**	0.192	**0.001**	0.49	1	1	1
pos. ha-sRNAs. antisense	counts	17	21	35	93	172	23	154	47	43	38	37
	enrichment	2.8	4.0	4.6	3.8	1.9	1.7	1.8	5.3	8.9	11.1	7.9
	p-values*	0.077	0.032	**0.012**	**0.003**	**0.005**	0.131	**0.005**	**0.004**	**0.002**	**0.001**	**0.003**
neg. ha-sRNAs. antisense	counts	73	53	103	265	977	142	365	70	70	33	66
	enrichment	3.7	4.1	5.2	4.5	3.5	4.5	2.7	6.8	8.6	6.3	10.2
	p-values*	**0.012**	**0.01**	**0.002**	**0**	**0**	**0.001**	**0.001**	**0.001**	**0.003**	**0.01**	**0.001**

*Significant enrichment highlighted by bold text (5% FDR. Benjamini-Hochberg). 0 equals a p-value of <0.001.
