# Supplementary material for: Parental Expression Variation of Small RNAs Is Negatively Correlated with Grain Yield Heterosis in a Maize Breeding Population
Source: Front Plant Sci. 2018 Jan 30;9:13. doi: 10.3389/fpls.2018.00013 (PMC5797689; doi:10.3389/fpls.2018.00013)
Supplement: Supplementary file 10 [file Table10.PDF]

Supplementary Material

Parental expression variation of small RNAs is negatively correlated with grain yield heterosis in a maize breeding population

Felix Seifert, Alexander Thiemann, Robert Grant-Downton, Susanne Edelmann, Dominika Rybka, Tobias A. Schrag, Matthias Frisch, Hugh G. Dickinson, Albrecht E. Melchinger, and Stefan Scholten*

Correspondence: Corresponding Author: stefan.scholten@uni-hamburg.de

Supplementary Table 10

Supplementary File S10 | Overlap of sRNAs between parental lines and hybrids at various expression thresholds.

		expression threshold [rpmqn]				
		**0.5**	**1**	**2**	**5**	**10**
# of distinct sRNAs		666233	261751	121462	48037	24446
fraction of sRNAs [%]	flint	21.34	17.90	15.98	14.08	13.45
	dent	12.24	9.71	8.54	7.54	6.92
	flint/dent	6.04	7.01	7.20	7.74	8.11
	hybrid	25.15	26.25	27.37	28.38	28.84
	flint/hybrid	7.31	6.55	5.62	5.06	4.93
	dent/hybrid	5.52	4.91	4.35	3.59	3.44
	flint/dent/hybrid	22.40	27.67	30.94	33.61	34.30
