# Supplementary material for: Parental Expression Variation of Small RNAs Is Negatively Correlated with Grain Yield Heterosis in a Maize Breeding Population
Source: Front Plant Sci. 2018 Jan 30;9:13. doi: 10.3389/fpls.2018.00013 (PMC5797689; doi:10.3389/fpls.2018.00013)
Supplement: Supplementary file 11 [file Image1.pdf]

Supplementary Material

Parental expression variation of small RNAs is negatively correlated with grain yield heterosis in a maize breeding population

Felix Seifert, Alexander Thiemann, Robert Grant-Downton, Susanne Edelmann, Dominika Rybka, Tobias A. Schrag, Matthias Frisch, Hugh G. Dickinson, Albrecht E. Melchinger, and Stefan Scholten*

Correspondence: Corresponding Author: stefan.scholten@uni-hamburg.de

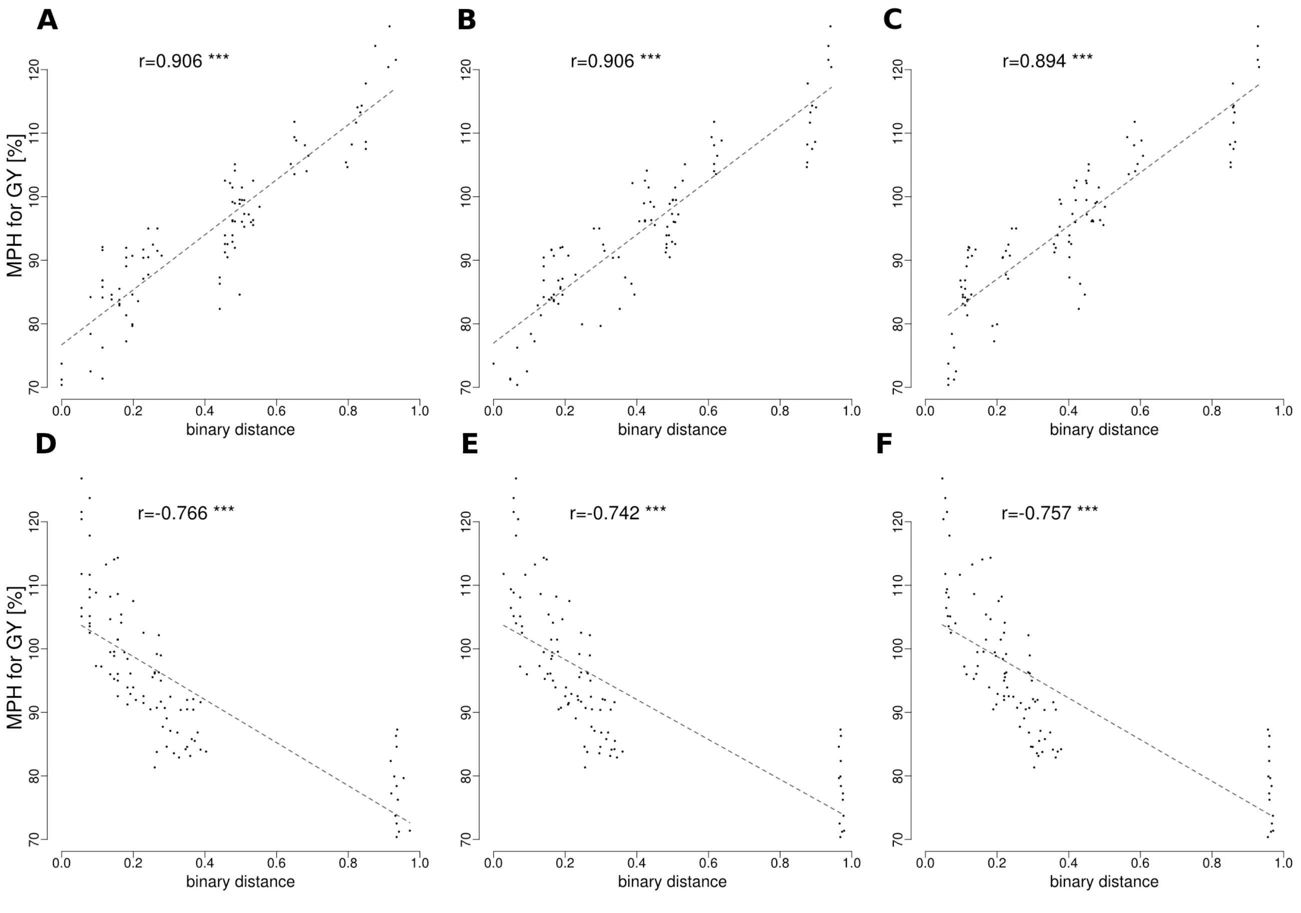

Supplementary Figure 1 | Separate classification of heterosis-associated sRNA by size classes. **(A-F)** Correlation of MPH for GY of the hybrids with the number of differentially expressed ha-sRNAs between inbred parents (binary distance) based on **(A-C)** positively **(A)** 21-nt, **(B)** 22-nt, **(C)** 24-nt, and **(D-F)** negatively **(D)** 21-nt, **(E)** 22-nt, **(F)** 24-nt associated ha-sRNAs (*** $p<0.001$).
