# Supplementary material for: Parental Expression Variation of Small RNAs Is Negatively Correlated with Grain Yield Heterosis in a Maize Breeding Population
Source: Front Plant Sci. 2018 Jan 30;9:13. doi: 10.3389/fpls.2018.00013 (PMC5797689; doi:10.3389/fpls.2018.00013)
Supplement: Supplementary file 12 [file Image2.pdf]

Supplementary Material

Parental expression variation of small RNAs is negatively correlated with grain yield heterosis in a maize breeding population

Felix Seifert, Alexander Thiemann, Robert Grant-Downton, Susanne Edelmann, Dominika Rybka, Tobias A. Schrag, Matthias Frisch, Hugh G. Dickinson, Albrecht E. Melchinger, and Stefan Scholten*

Correspondence: Corresponding Author: stefan.scholten@uni-hamburg.de

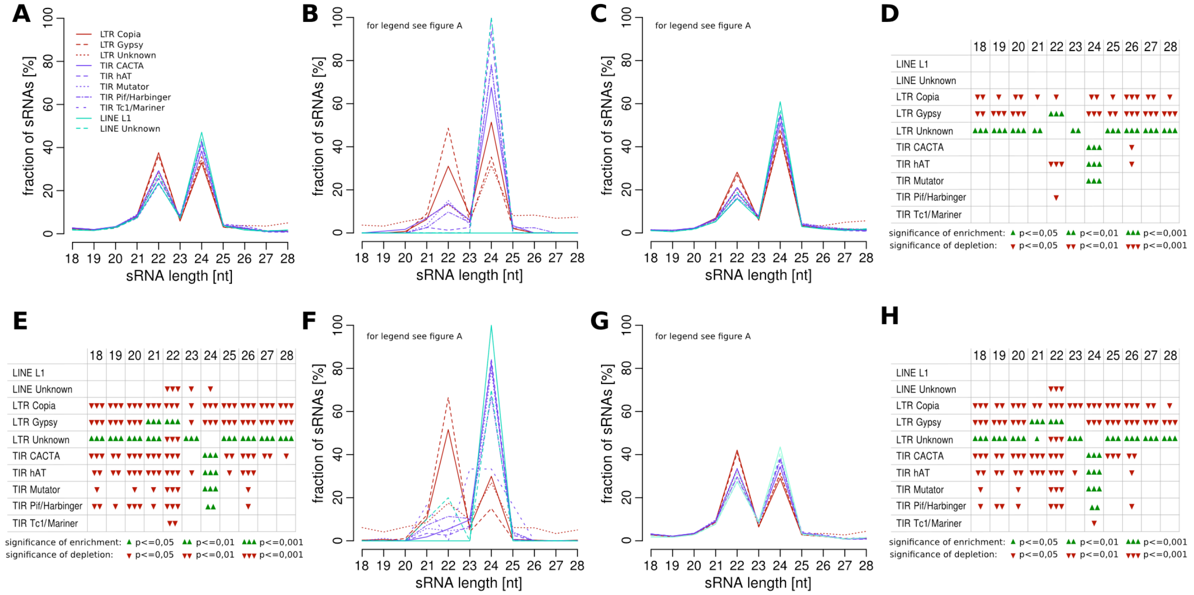

Supplementary Figure 2 | Distribution of ha-sRNAs to repeat super-families. (**A**) Average bootstrap size distribution of sRNAs mapped to repeat super-families (1000 runs), with identical size distribution as all ha-sRNAs. (**B**) Size distribution of positively ha-sRNAs mapped to repeat super-families. (**C**) Average bootstrap size distribution of sRNA mapped to repeat super-families (1000 runs), with identical size distribution as positively ha-sRNAs. (**D**) Significances of enrichment/depletion for positively ha-sRNAs mapping to repeat super-families. (**E**) Significances of enrichment/depletion for all ha-sRNAs mapping to repeat super-families. (**F**) Size distribution of negatively ha-sRNAs mapped to repeat super-families. (**G**) Average bootstrap size distribution of sRNA mapped to repeat super-families (1000 runs), with identical size distribution as negatively ha-sRNAs. (**H**) Significances of enrichment/depletion for negatively ha-sRNAs mapping to repeat super-families.
