# Supplementary material for: Parental Expression Variation of Small RNAs Is Negatively Correlated with Grain Yield Heterosis in a Maize Breeding Population
Source: Front Plant Sci. 2018 Jan 30;9:13. doi: 10.3389/fpls.2018.00013 (PMC5797689; doi:10.3389/fpls.2018.00013)
Supplement: Supplementary file 13 [file Image3.pdf]

## *Supplementary Material*

# Parental expression variation of small RNAs is negatively correlated with grain yield heterosis in a maize breeding population

**Felix Seifert, Alexander Thiemann, Robert Grant-Downton, Susanne Edelmann, Dominika Rybka, Tobias A. Schrag, Matthias Frisch, Hugh G. Dickinson, Albrecht E. Melchinger, and Stefan Scholten***

**Correspondence:** Corresponding Author: stefan.scholten@uni-hamburg.de

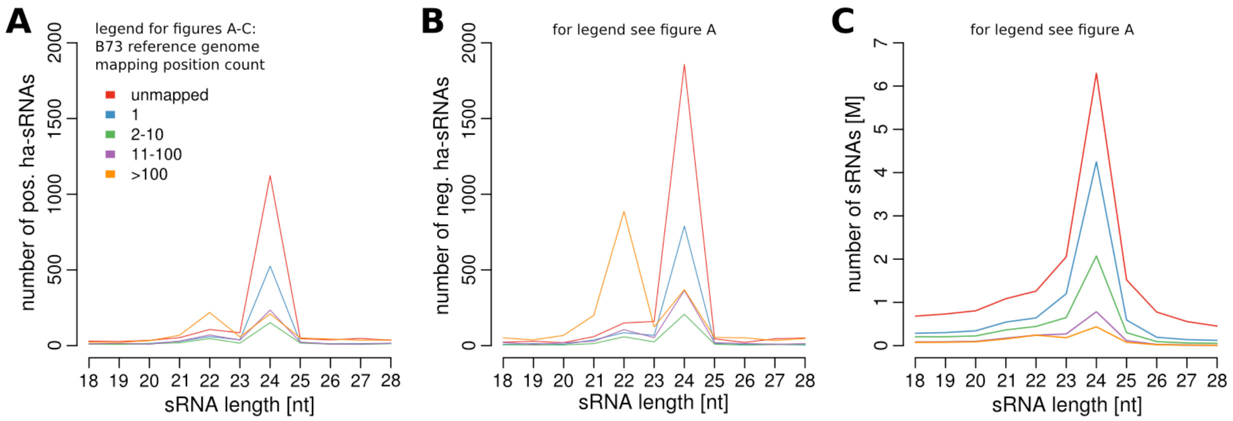


**Supplementary Figure 3 | Mapping count frequencies of sRNAs to the B73 reference genome.** Mapping count frequencies of **(A)** positively ha-sRNAs, **(B)** negatively ha-sRNAs, (**C**) all sRNAs, separately for sRNA of different length.
